# Two Doses of Candidate TB Vaccine MVA85A in Antiretroviral Therapy (ART) Naïve Subjects Gives Comparable Immunogenicity to One Dose in ART+ Subjects

**DOI:** 10.1371/journal.pone.0067177

**Published:** 2013-06-28

**Authors:** Tandakha N. Dieye, Birahim P. NDiaye, Alle B. Dieng, Marema Fall, Nathaniel Britain, Samantha Vermaak, Makhtar Camara, Halimatou Diop-Ndiaye, Ndeye Fatou Ngom-Gueye, Papa A. Diaw, Coumba Toure-Kane, Papa S. Sow, Souleymane Mboup, Helen McShane

**Affiliations:** 1 Laboratoire de Bacteriolgie-Virologie, Centre Hospitalier Universitaire Le Dantec, Dakar, Senegal; 2 Centre de Recherche Clinique, Centre Hospitalier Universitaire de Fann, Dakar, Senegal; 3 Jenner Institute, University of Oxford, Oxford United Kingdom; 4 Centre de Traitement Ambulatoire, Centre Hospitalier Universitaire de Fann, Dakar, Senegal; University of Palermo, Italy

## Abstract

Tuberculosis (TB) is a global public health problem exacerbated by the HIV epidemic. Here we evaluate a candidate TB vaccine, MVA85A, in a Phase I study in HIV-infected adults in Senegal. 24 patients were enrolled: Group 1∶12, antiretroviral therapy (ART) naïve, adults, with CD4 counts >300 and HIV RNA load <100 000 copies/ml. Group 2∶12 adults, stable on ART, with CD4 counts >300, and an undetectable HIV RNA load. Safety was evaluated by occurrence of local and systemic adverse events (AEs) and by monitoring of CD4 count, HIV RNA load, haematology and biochemistry. Immunogenicity was evaluated by *ex-vivo* interferon-gamma ELISpot assay. 87.7% of AEs were mild; 11.6% were moderate; and 0.7% were severe. 29.2% of AEs were systemic; 70.8% were expected local AEs. There were no vaccine-related Serious Adverse Events (SAEs) or clinically significant effects on HIV RNA load or CD4 count. In ART naive subjects, the first MVA85A immunisation induced a significant immune response at 1 and 4 weeks post-immunisation, which contracted to baseline by 12 weeks. Durability of immunogenicity in subjects on ART persisted out to 24 weeks post-vaccination. A second dose of MVA85A at 12 months enhanced immunogenicity in ART naïve subjects. Subjects on ART had higher responses after the first vaccination compared with ART naïve subjects; responses were comparable after 2 immunisations. In conclusion, MVA85A is well-tolerated and immunogenic in HIV-infected subjects in Senegal. A two dose regimen in ART naïve subjects is comparable in immunogenicity to a single dose in subjects on ART.

Clinicaltrials.gov trial identifier NCT00731471.

## Introduction

Tuberculosis (TB) is one of the leading causes of death from a single infectious agent. One third of the world’s population is latently infected with *Mycobacterium tuberculosis* (*M. tb*) [1] and TB kills about 1.45 million people annually worldwide [2]. Latently infected individuals have a 10% lifetime risk of developing active TB disease, or a 10% annual risk if they become immunosuppressed [3]. TB prevention remains one of today's greatest public health challenges and an efficacious vaccination strategy will be an essential tool to control it. The highest numbers of TB cases are in Africa and South East Asia. Even though TB prevalence is decreasing in all six WHO regions [2], the burden in higher incidence countries is in part due to the increasing prevalence of HIV [4], [5].

HIV likely serves as a driver of TB at the population level by increasing the incidence of TB and TB- related deaths in a population of immunodeficient individuals susceptible to both primary and reactivation TB [6]. The relative risk of TB doubles in the first year after HIV infection, when CD4 counts are still preserved, and continues to increase during the years after seroconversion as CD4 counts decrease [7]. In individuals with latent *M. tb* infection, HIV accelerates and augments progression to reactivation of TB. The initiation of ART can reduce the risk of HIV-associated TB by restoring the immune response to *M. tb*, leading to sustained reductions in long-term TB risk [8]. However, the initial weeks of immune recovery may be associated with a transiently heightened risk of TB [9]. Thereafter the risk of TB decreases rapidly particularly in the first 2–3 years of ART [10]. However, even for patients on ART who have CD4 counts above 500 cells/µl, the TB incidence rate can still remain 2-fold higher than those for adults without HIV [8].

The only available vaccine, *Mycobacterium bovis* BCG, is largely ineffective at protecting against adult pulmonary disease, but does protect against disseminated TB and tuberculosis meningitis in children [11], [12]. BCG is contraindicated in HIV-infected infants, even in settings where TB is highly endemic, and a safer, more effective vaccine is urgently needed [13]. There are many new TB vaccines at different stages of development [14]. MVA85A is a candidate TB vaccine designed to enhance BCG [15], [16]. It is a subunit-viral vectored vaccine that uses Modified Vaccinia virus Ankara as a delivery system for the mycobacterial antigen 85A [17]. MVA85A boosts both antigen specific IFN-γ secreting CD4+ and CD8+ T cells and can induce higher levels of protection against aerosol challenge than after BCG alone in mice, non-human primates and cattle [18]–[20]. The promising safety and immunogenicity of this candidate vaccine in UK trials has led to further Phase I and IIa clinical trials in target populations in South Africa, The Gambia and Senegal [15], [21], [22]. The immunogenicity of MVA85A has been well characterised and the immunity induced is thought to be important for protection. A recent phase IIb safety and efficacy trial in BCG-vaccinated South African infants demonstrated MVA85A to be safe but not to enhance BCG-induced protective immunity [23]. The level of antigen-specific CD4+ T cells induced in this infant trial was modest. It is not clear whether, in populations where the immunogenicity is greater; this vaccine would confer significant protection [24]. In adults, high frequencies of antigen-specific IFN-γ-producing polyfunctional CD4+ T cells are induced by MVA85A, including expansion of a memory population, and the frequency of antigen-specific cells remains significantly higher than baseline for at least one year after vaccination [25]. HIV-infected subjects are a really important target population for a new TB vaccine and MVA85A has been safely administered to HIV-infected subjects in the UK and South Africa [26], [27].

In this study, we evaluated for the first time the safety and immunogenicity of MVA85A in Senegalese HIV-infected subjects, and furthermore evaluated both comparative immunogenicity in subjects on and off anti-retroviral therapy, and whether a second, homologous boosting immunisation could improve the elicited immune response in HIV-1-infected individuals.

## Materials and Methods

### Study Population

24 healthy HIV-1 infected volunteers, male and female, aged 18 to 50 years were enrolled in the study after a screening visit at the Ambulatory Treatment Centre (CTA) and the Centre Régional de Recherche Clinique et de Formation at the Fann Teaching hospital between August 2008 and February 2010 **(** **Table 1** **)**. All subjects gave written informed consent. The trial was approved by the Comité National d'Ethique pour la Recherche en Santé (CNERS) in Senegal and the Oxford Tropical Research Ethics Committee (OXTREC) in the UK. The protocol for this trial and supporting CONSORT checklist are available as supporting information (see Checklist S1 and Protocol S1).

**Table 1:** Epidemiological and clinical characteristics of HIV-1 eligible volunteers.

Characteristics	HIV+ART– Group 1 (n = 12)	HIV+ART+ Group 2 (n = 12)
**Age**, years (Min-Max)	35 (24–44)	35 (30–47)
**Sex**		
Male	2	4
Female	10	8
**Hemoglobin (g/dl)**	11.9 (10.1–15.5)	12.45(11.6–15.5)
**Neutrophils (10^9^/l)**	1.6 (1–2.3)	1.8 (1–2.9)
**Lymphocytes (10^9^/l)**	1.6 (1.1–2.8)	1.75 (1–2.6)
**ALAT (UI/l)** *	18 (9–49)	14 (10–50)
**CD4 counts/µl**	572 (300–1120)	543 (440–1281)
**HIV RNA load copy/ml**	3089 (40–95221)	40
**ART therapy**	No	Yes
**M. tb infection**	7 (58.3%)	12 (100%)

Data are median (interquartile range) values when indicated.

* ALAT: alanine transferase.

**Figure 1:**
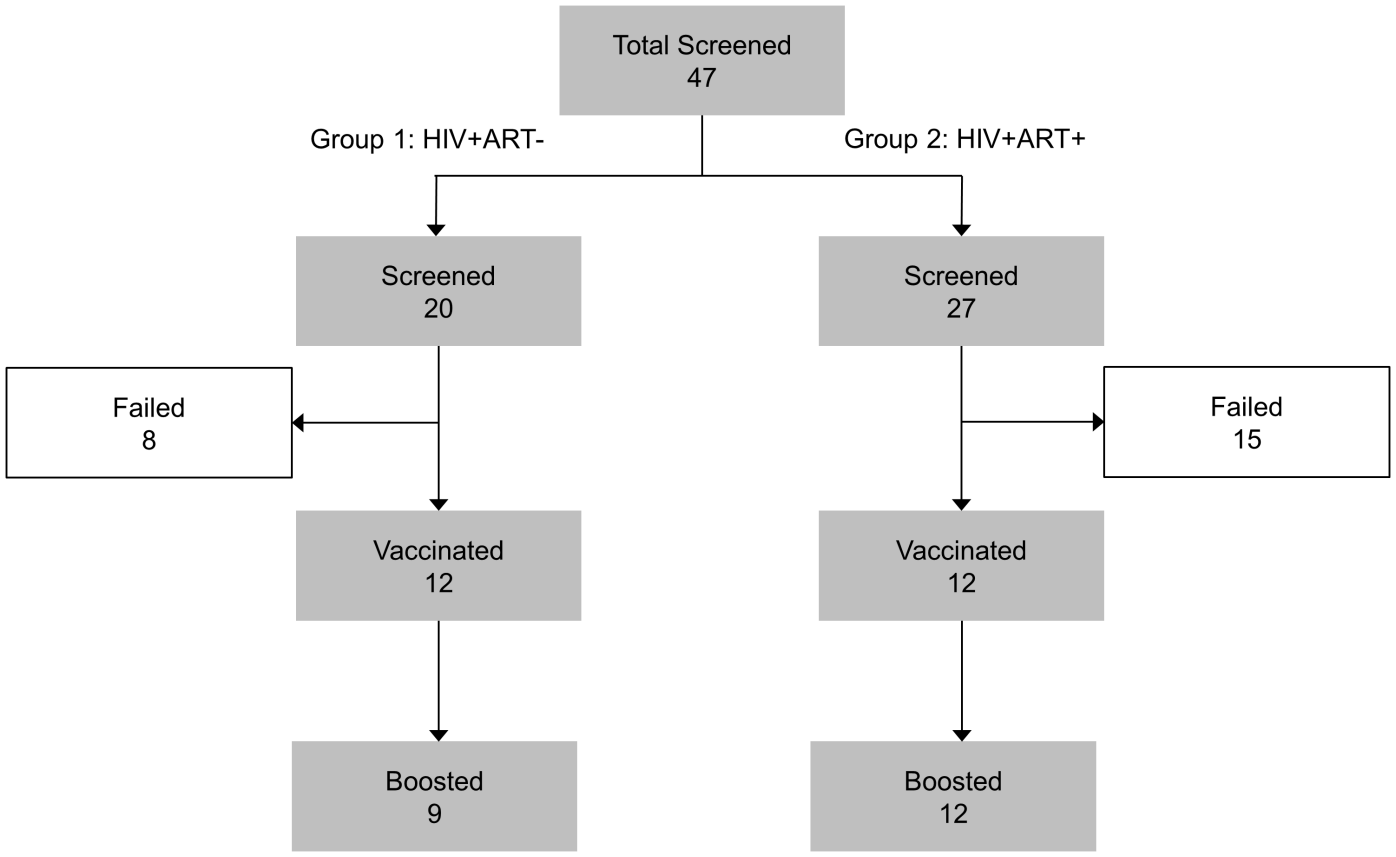
Consort diagram of the study population.

Eligible subjects attended the Bacteriologie et Virologie Laboratoire at Le Dantec Teaching Hospital (LBV) for vaccination **(** **Figure 1** **).** In group 1, twelve patients were HIV-1 antibody positive; diagnosed at least 6 months previously; had CD4+ T cell counts >300 and CD4 count nadir 300; HIV RNA load ≤100,000 copies/ml and were not on ART. In group 2, twelve patients were HIV-1 antibody positive diagnosed at least 12 months previously, had CD4 counts prior to enrolment >300 and CD4 count nadir >100, were stable on the same ART regimen for at least 12 months, and had had undetectable HIV RNA loads (<75 copies/ml) for at least 12 months.

Exclusion criteria were focused on clinically significant abnormal findings from screening biochemistry or haematology, urinalysis, previous history of TB disease and/or treatment, any AIDS defining illness, prior receipt of a recombinant MVA85A, and chronic administration (more than 14 days) of immunosuppressive drugs. Reasons for exclusion are shown in **Table 2** **.**

**Table 2:** Reasons for exclusion from recruitment into the trial.

	Number of volunteers (% of those screened*)
**Total screened**	**47 (100)**
**Enrolled**	**24 (51)**
**Excluded**	**23 (49)**
Abnormal laboratory findings	7 (15)
Hepatitis B or C positive	5 (11)
HIV RNA load >100,000 copies/ml (ART naïve group 1)	5 (11)
CD4 count <300	4 (9)
Unavailable in study period	2 (4)
Previous medical history	1 (2)
Pregnant	1 (2)
Not required (recruitment full)	1 (2)

* Some subjects were excluded for more than one reason.

HIV status was evaluated in plasma by current testing combining ELISA and Western Blotting assays. Absolute CD4+ and CD8+ cells were assayed from fresh blood samples following our routine procedure on the FACSCount™ flow cytometer (Becton Dickinson Biosciences, San Jose, CA).

The plasma HIV-1 RNA load was measured using the Abbott m2000 RealTime HIV-1 assay following manufacturer’s instruction (Abbott Molecular, Des Plaines IL USA). Briefly, the Abbott RealTime HIV-1 assay is an in vitro reverse transcription-polymerase chain reaction (RT-PCR) assay for the quantitation of HIV-1 on the automated m2000 System or with m24sp extraction in human plasma from HIV-1 infected individuals over the range of 40 to 10,000,000 copies/mL (5 log_10_ copies). The trial was registered on clinicaltrials.gov (number NCT00731471).

### Vaccination Procedures and Follow Up Visit

All eligible subjects received two intradermal injections of 1×10^8^ pfu MVA85A, one at Day 0 and a booster vaccine approximately 6–12 months later. Eligibility was re-verified prior to the second immunisation; two subjects were excluded from boost vaccination according to the exclusion criteria and one due to pregnancy. Subjects stayed in the clinical trial unit at the Bacteriologie et Virologie Laboratoire at Le Dantec Teaching Hospital (LBV) for 60 minutes (+/−10 mins) after vaccination for observation. After each immunisation, subjects were followed-up at weeks 1, 4, 12, and 24. At each follow-up visit, all AEs occurring in participants were reported for the clinical safety assessment. Blood was collected to assess the vaccine biological safety profile (biochemistry, haematology, CD4 count and HIV RNA load) and immunogenicity.

### Isolation of Peripheral Blood Mononuclear Cells (PBMC)

Briefly, 20–30 ml of blood was drawn into sodium heparin vacutainer tubes. The PBMC were isolated by density gradient centrifugation at 400**×** g for 35 mins using Ficoll-Hypaque plus (Amersham, Biosciences, USA). The cells were washed in RPMI 1640 using a falcon tube to total volume of 50 ml. Cells were re-suspended in 1 ml of complete culture medium consisting of RPMI 1640 medium supplemented with 10% FCS, 2 mM L-glutamine, 1 mM sodium puryvate, 100 U/ml penicillin and 100 µg/ml streptomycin, (all Sigma Aldrich, Steinheim, Germany).

### ELISpot Assay

The immunogenicity of MVA85A was determined by measuring the frequency of IFN-γ spot-forming units (SFUs) by *ex-vivo* ELISpot assay using the ELISpot Kit (Mabtech, Besancon, France) as previously described [28]. ELISpot plates (Millipore, Tullagreen, Carrigtwohill, Ireland) were coated with 50 µl of anti IFN-γ Ab at a final concentration of 15 µg/ml in the carbonate-bicarbonate buffer 0.5M. After overnight incubation, plates were washed 5 times in sterile phosphate buffered saline (PBS) and blocked by adding 100 µl of complete culture medium to each well and incubating for 2–5 h at 37°C. After discarding the blocking solution the isolated PBMCs, in a concentration of 0.3 x 10^6^ PBMC/100 µl were added to duplicate wells and incubated in the presence of the antigen or controls. Recombinant antigen 85A (Lionex) was used at a final concentration of 10 µg/ml. Peptides (Peptide Protein Research, UK) were used as a single peptide pool of 66 15mers which spanned the length of antigen 85A (overlapping by 10 amino acids; at 2 µg/ml each), and as 7 summed pools of 9–10 overlapping 15mer peptides. *M. tb* PPD (purified protein derivative; 10 µg/ml; SSI) was also used as a recall antigen. ESAT-6/CFP10 (Peptide Protein Research, UK) peptides pools were added at the final concentration of 10 µg/ml to detect latent *M. tb* infection. Medium alone served as a negative control, and phytohemagglutinin (10 µg/ml; Sigma) was the positive control.

ELISpot plates were incubated for 18–20 hours at 37°C and then washed 5 times with PBS/0.05% Tween20. 50 µl of diluted biotin anti-IFN-γ AB 1/1000 in PBS was added and incubated for 1 hour and then washed 5 times with PBS 0.05% Tween 20. Finally, 50 µl of developing buffer (BIO-RAD, UK) was added to each well and incubated 5–10 minutes at room temperature until distinct spots developed. ELISpot plates were washed 3 times in tap water, dried overnight and read using the AID ELISpot reader (Strassberg, Germany).

The number of IFN-γ-secreting cells (ISC) was standardised per 10^6^ PBMC. The response to each epitope or antigen was considered positive if the number of ISC was *i)* greater than twice the response without antigen stimulation, after deduction of the background level with unstimulated cells, and *ii)* at least 5 spots more than the negative control well, and *iii)* two negative control wells containing only complete medium and PBMC had less than 20 spots per well, and *iv)* if the counts were >15 spots/well, the count between duplicates could not vary by more than 50% in a total of 90% of all wells.

### Statistical Analysis

The data were analysed using the GraphPad Prism software version 5 statistical package (Software MacKiev, GraphPad, San Diego, CA). For non-normally distributed data the Mann-Whitney *U* test was used to compare two groups and *P* < 0.05 was considered significant. All tests of significance were two tailed. Comparison of *M. tb* infection rates between the two groups was done using a Fisher’s exact test.

## Results

### Characteristics of the Study Population

All study groups were similar in parameters shown on **Table 1** at screening, except for the HIV RNA load and anti-retroviral treatment (ART). Hepatitis markers, HBS antigen, anti-HB core and hepatitis C antibodies were negative for all HIV-1 infected volunteers.

Using the ESAT/CFP10 *ex-vivo* ELISpot, 58.3% (7/12) of HIV+ART**–** volunteers (group 1) were latently infected with *M. tb* and all HIV+ART+ volunteers (group 2) were identified as *M. tb* infected (p = 0.0373).

### MVA85A Clinical Safety Data

Most of the AEs (87.7%; 264/301) were mild in severity; 11.6% (35/301) were moderate and the remaining 0.7% (2/301) were severe. Systemic AEs made up 29.2% (88/301) of the total, and symptoms included headaches, fever, fatigue, and/or joint pain, all of which spontaneously resolved. 70.8% (213/301) of AEs were expected local AEs. There were no vaccine-related SAEs observed during the study.

### MVA85A Biological Safety Data

#### Hematology and Biochemistry parameters

Only one HIV+ART**–** volunteer had a transient neutropenia at week 1 post booster dose, where the neutrophil count dropped from 1.5 x 10*9/l to 0.6 x 10*9/l and recovered at 12 weeks. This was not considered clinically significant and the patient remained asymptomatic throughout.

#### CD4 counts and HIV RNA load monitoring

**Figure 2:**
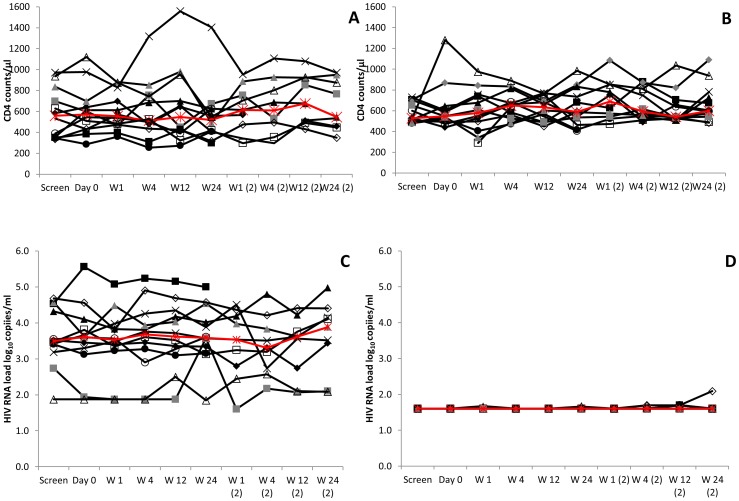
CD4+ T cell responses and HIV RNA load measurements after MVA85A vaccination. (A) CD4+ T cell count in 12 HIV+ART−, volunteers (group 1) and (B) in 12 HIV+ART+ volunteers (group 2). (C) HIV RNA load in 12 HIV+ART**–** volunteers (group 1) and (D) in 12 HIV+ART+ volunteers (group 2). Median values shown in red.

CD4 counts remained stable during the study **(** **Figure 2A&B** **).** There were no clinically significant changes in CD4 count throughout this study.

Median CD4 count/µl was similar in the HIV+ART**–** group at day 0 at the first (572) and at the booster dose (658); p = 0.4. Similar CD4 counts were observed in the HIV+ART+ group at day 0 at the first vaccination (543) and at the booster dose (587); p = 0.7.

No clinically significant changes in HIV RNA load were seen in any of the 24 subjects **(** **Figure 2C, 2D** **).** All HIV+ART+ volunteers had undetectable HIV RNA load levels except one individual who had a detectable HIV RNA load (123 copies/ml) at week 24 post second vaccination. This was not considered clinically significant.

#### MVA85A immunogenicityMVA85A-specific IFN-γ-producing T cell responses in the HIV+ART**–** group

A significant increase in Ag85A CD4+ T cells to peptides (pooled and single) and recombinant antigen was found after the first vaccination in the HIV+ART**–** group. At 4 weeks this response was still higher than baseline (p = 0.04). At 12 weeks post vaccination, only the summed pooled peptide response remained higher than baseline (p = 0.046). At six months the summed peptide pool response was not significantly above baseline (p = 0.26) **(** **Table 3** **)**.

**Table 3:** IFN-γ secreting cells (ISC) with the sum of peptides (A), the single pool (B) per 10^6^ PBMCs to the HIV+ART**–** group and HIV+ART+ group at the baseline timeline versus post vaccination.

A				*Summed peptide pools*				
					Day 0 *vs* W1	Day 0 *vs* W4	Day 0 *vs* W12	Day 0 *vs* W24
**HIV+ART– Group** (first dose)	**Median**				31 vs 901	31 vs202	31 vs 108	31 vs 84
	***P*** *				*(<0.0003)*	*(<0.05)*	*(<0.04)*	*(0.2)*
**HIV+ART– Group** (second dose)	**Median**				31 vs 1517	31 vs 4358	31 vs 526	31 vs 212
	***P*** *				*(<0.0001)*	*(<0.0001)*	*(<0.0001)*	*(<0.0008)*
**HIV+ART+ Group** (first dose)	**Median**				446 vs 3146	446 vs 2693	446 vs 1494	446 vs 997
	***P*** *				*(<0.0001)*	*(<0.0003)*	*(<0.01)*	*(<0.03)*
**HIV+ART+ Group** (second dose)	**Median**				446 vs 3339	446 vs 2662	446 vs 1444	446 vs 1131
	***P*** *				*(<0.002)*	*(<0.0001)*	*(<0.003)*	*(<0.0007)*
**B**			***Single pool peptides***					
		**Day 0 ** ***vs*** ** W1**				**Day 0 ** ***vs*** ** W4**	**Day 0 vs W12**	**Day 0 ** ***vs*** ** W24**
**HIV+ART– Group** (first dose)	**Median**		8 vs 423			8 vs 50	8 vs 21	8 vs 23
	***P*** *		*(<0.0001)*			*(<0.06)*	*(0.1)*	*(0.1)*
**HIV+ART– Group** (second dose)	**Median**		8 vs 478			8 vs 597	8 vs 140	8 vs 133
	***P*** *		*(<0.0001)*			*(<0.0001)*	*(<0.001)*	*(<0.007 )*
**HIV+ART+ Group** (first dose)	**Median**		68 vs 580			68 vs 317	68 vs 233	68 vs 181
	***P*** *		*(<0.0004)*			*(<0.0002)*	*(<0.01)*	*(<0.05)*
**HIV+ART+ Group** (second dose)	**Median**		68 vs 389			68 v 428	68 vs 196	68 vs 220
	***P*** *		*(<0.0009)*			*(<0.002)*	*(<0.01)*	*(<0.004)*

* Mann Whitney test.

After the second vaccination the HIV+ART– group had a significant increase in Ag85A CD4+ T cell response to peptides (pooled and single) and recombinant antigen. At week 24 this response remained significantly above baseline (recombinant antigen, p = 0.014; single peptide pool p = 0.0075; summed peptide pool p = 0.0008) **(** **Table 3** **)**.

**Figure 3:**
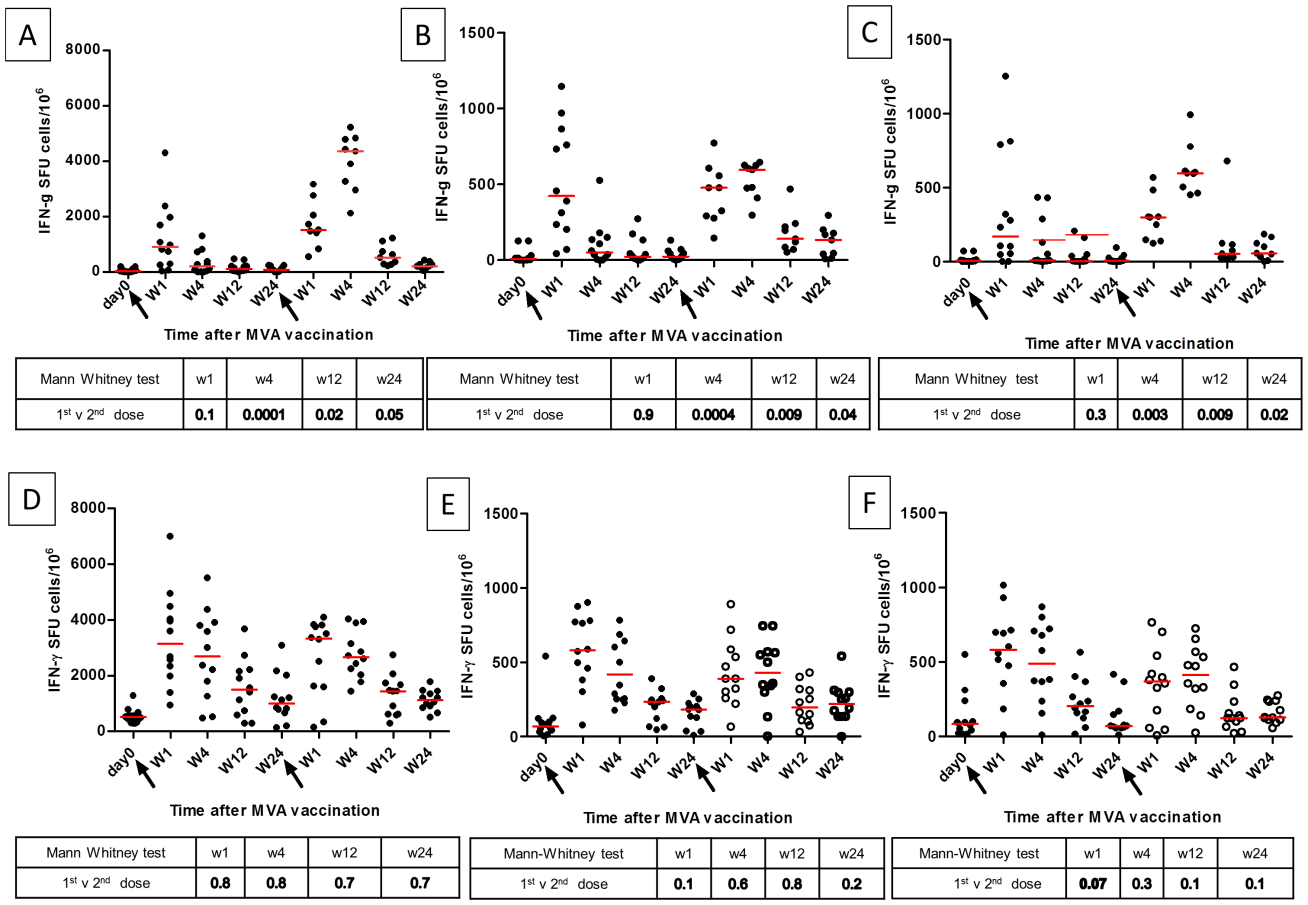
Immunogenicity of MVA85A – HIV+ART– group (A, B, C) compared to HIV+ART+ group (D, E, F). Measured by ex vivo ELISpot assay in the sum of pool of antigen 85A peptides **(A, D)**, the single pool of 66 peptides antigen 85A **(B, E)** and in the recombinant antigen 85A **(C, F)**. The figures also compare the responses after the first (arrow) and second vaccination (arrow).

When we compared the magnitude of IFN-γ producing T cell responses after the first and second vaccination, the response was not significantly different at 1 week with the sum of pooled peptide, the single 66 peptides, or the recombinant 85A antigen (p = 0.30; 0.80 and 0.35 respectively) **(** **Figure 3A, 3B, 3C** **)**. However at week 4, the response after the second dose was significantly higher than after the first with the sum of pooled peptides (p = 0.0039), the single 66 peptides (p = 0.0039) and the recombinant 85A antigen (p = 0.0039) **(** **Figure 3A, 3B, 3C** **)**. At 12 weeks, the response remained significantly higher after the second dose when compared to the first for the sum of total peptides (p = 0.023) and the single pooled peptides (p = 0.043) **(** **Figure 3A, 3B** **)** but not with recombinant antigen (p = 0.20). At 24 weeks post vaccination, the response remained higher after the second vaccination compared to the first for summed peptide pools (p = 0.030) the single peptide pool (p = 0.027) and with recombinant antigen (p = 0.0078) **(** **Figure 3A, 3B, 3C** **)**.

#### MVA85A-specific IFN-γ-producing T cell responses in the HIV+ART+ group

Baseline responses in the HIV+ART+ group were higher than in the HIV+ART**–** group for all antigens (summed peptide pool p = 0.0001; single peptide pool p = 0.01; recombinant antigen p = 0.0004). In the HIV+ART+ group, there was a significant increase in Ag85A CD4+ T cell response to peptides (pooled and single) and recombinant antigen after the first and second vaccination. At 24 weeks this response remained higher than baseline after both first and second vaccination. IFN-γ-producing T cell responses were significantly higher than baseline at week 1, 4, 12 and 24 but no variation was found when we compared the first vaccination to the second vaccination. There was no difference in magnitude of responses after the first and second immunisation in HIV+ART+ volunteers **(** **Figures 3D–F** **)**.

#### ELISpot responses of HIV+ART– versus HIV+ART+ groups after the first vaccination

The baseline pre-vaccination responses to PPD, recombinant 85A and the peptide pools were higher in the HIV+ART+ group than in the HIV+ART**–** group **(** **Table 4A, 4B** **)**. After vaccination, immune responses to all antigens were significantly higher in the HIV+ART+ group than in the HIV+ART**–** group for all time points except week 1 post-vaccination where the variance is higher with the single pool (p = 0.4)**(** **Table 4A, 4B** **)**.

**Table 4:** IFN-γ secreting cells (ISC) with the summed peptide pools (A, C), the single pool (B, D) per 10^6^ PBMCs for the HIV+ART**–** and HIV+ART+ groups after the first dose and second dose of MVA85A.

A		1^st^ Dose							
*Summed peptide pools*		Day 0	W1		W4		W12		W24
**HIV+ART– Group**	**Median**	31	901		202		108		84
	**(Min-Max)**	(0–195)	(46–4298)		(10–1305)		(9–476)		(7–244)
**HIV+ART+ Group**	**Median**	446	3142		2693		1494		997
	**(Min-Max)**	(289–1281)	(948–6996)		(478–5508)		(290–3677)		(140–3081)
***P*** *		***0.0001***	***0.002***		***0.0002***		***0.0001***		***0.0003***
**B**		**1^st^ Dose**							
***Single pool peptides***		**Day 0**	**W1**		**W4**		**W12**		**W24**
**HIV+ART− Group**	**Median**	8	423		50		21		23
	**(Min-Max)**	(0–127)	(43–1147)		(0–527)		(0–273)		(0–132)
**HIV+ART+ Group**	**Median**	68	580		317		233		181
	**(Min-Max)**	**(**4–539**)**	**(**78–900**)**		**(**176–782**)**		**(**47–389**)**		**(**9–288**)**
***P*** *		***0.01***	***0.4***		***0.003***		***0.002***		***0.003***
**C**		**2^nd^ Dose**							
***Sum pool peptides***		**W1**		**W4**		**W12**		**W24**	
**HIV+ART− Group**	**Median**	1517		4358		526		212	
	**(Min-Max)**	(547–3172)		(2125–5225)		(225–1220)		(57–431)	
**HIV+ART+ Group**	**Median**	3339		2662		1444		1131	
	**(Min-Max)**	(159–4096)		(1440–4033)		(296–2746)		(510–1793)	
***P*** *		***0.07***		***0.01***		***0.01***		***0.0001***	
**D**		**2^nd^ Dose**							
***Single pool peptides***		**W1**		**W4**		**W12**		**W24**	
**HIV+ART− Group**	**Median**	478		597		140		133	
	**(Min-Max)**	(145–773)		(297–646)		(53–469)		(9–296)	
**HIV+ART+ Group 2**	**Median**	389		428		196		220	
	**(Min-Max)**	(67–889)		(3–745)		(30–429)		(0–539)	
***P*** *		***0.5***		***0.3***		***0.5***		***0.1***	

Mann Whitney test was used.

#### ELISpot responses of HIV+ART− versus HIV+ART+ groups after the second vaccination

Post second vaccination, immune responses to the summed peptide pools were significantly higher in the HIV+ART+ group compared to the HIV+ART− group at week 12 and week 24 **(** **Table 4C** **)**, but immune responses to the recombinant antigen and single peptide pools were comparable **(** **Table 4C, 4D** **)**.

#### ELISpot responses of HIV+ART− group after second vaccination versus HIV+ART+ group after the first vaccination

Comparing immune responses after the second vaccination in the HIV+ART− group to the first vaccination in the HIV+ART+ group, post vaccination responses to summed peptide pools were higher in the HIV+ART+ group than in the HIV+ART− group **(** **Table 5A** **).** Responses to the single peptide pool antigen were comparable between groups **(** **Table 5B** **)**.

**Table 5:** IFN-γ secreting cells (ISC) with the summed peptide pools (A), the single pool (B) per 10^6^ PBMCs for the HIV+ART− group (second dose) versus the HIV+ART+ group (first dose).

*A Summed peptide pools*			W1	W4	W12	W24
HIV+ART− Group	Median	1517		4358	526	212
***2^nd^ dose***	**(Min-Max)**		(547–3172)	(2125–5225)	(225–1220)	(57–431)
**HIV+ART+ Group**	**Median**		3142	2693	1494	997
***I^st^ dose***	**(Min-Max)**		(948–6996)	(478–5508)	(290–3677)	(140–3081)
***P*** *			***0.03***	***0.07***	***0.01***	***0.005***
**B ** ***Single pool peptides***			**W1**	**W4**	**W12**	**W24**
**HIV+ART**− **Group**	**Median**		478	597	140	133
***2^nd^ dose***	**(Min-Max)**		(145–773)	(297–646)	(53–469)	(9–296)
**HIV+ART+ Group**	**Median**		580	317	233	181
***1^st^ dose***	**(Min-Max)**		(78–900)	(176–782)	(47–389)	(9–288)
***P*** *			***0.1***	***0.2***	***0.3***	***0.3***

* Mann Whitney test.

## Discussion

MVA85A was found to be well tolerated in HIV-infected subjects in Senegal, consistent with findings from a UK study in HIV-infected subjects [26]. AEs were consistent with previously published trials [26], [29]. No clinically significant effects on either HIV RNA load or CD4 count were observed in this study.

The magnitude of the antigen 85A-specific immune response was high one week post vaccination, but contracted quickly and was no longer above baseline 4 weeks after vaccination in the ART naïve HIV+ART− group 1. A previous study using MVA85A in HIV negative adult volunteers in The Gambia show a better frequency in IFN-γ specific response against 85A peptides and this amplitude was maintained after 12 weeks [21]. This strong immune response in a genetically and mycobacteriologically similar population suggests that the reduced durability seen in this study is related to chronic HIV- infection. Others have shown that CD4+ T cells from HIV infected individuals failed to secrete IFN-γ by ex vivo ELISpot after stimulation with Hepatitis B, Hepatitis A and tetanus toxoid [30]. These naive CD4+ T cells are the subset most reduced in chronic HIV infection, and this frequently results in a loss of CD4 cellular and humoral specific immune response to HIV, pathogens and vaccines [31]–[33].

Importantly, the administration of a second dose of MVA85A in HIV-1 positive individuals, 12 months after the first immunisation, resulted in a significantly stronger effector response that was maintained out to 24 weeks post vaccination. This suggests that a booster dose may be necessary in ART naïve, HIV infected people. Although the proportion of *M. tb* infected subjects differed between the two groups, previous studies have shown no difference in T cell immunogenicity after MVA85A when comparing BCG vaccinated and *M. tb* infected subjects [27], [34]. Current standard of care in Senegal is to commence ARTs when a patient’s CD4+ T cell count is less than 350, in line with guidelines throughout the rest of the world.

ART reduces the incidence of TB disease in HIV-infected adults by up to 90% [35]. TB is the predominant disease associated with immune reconstitution inflammatory syndrome (IRIS) seen during the early stage of ART [8]. This phenomenon has been estimated to account for approximately 40% of TB cases presenting during early ART, however, the risk decreases rapidly in the first 2–3 years of ART [10]. Based on the important role of Th1 cells in TB, it may be more effective to vaccinate healthy HIV-1 individuals who have their immune response reconstituted by ART. Increased levels of naïve CD4 counts in blood correlate to total CD4 cells after starting ART because cells are preferentially depleted in lymphoid tissues during HIV infection [36], [37]. In this study, the immune responses post vaccination with MVA85A in subjects stable on ART were significantly higher than those seen in subjects not on ART, despite controlling for CD4 count. Furthermore, a single dose of MVA85A in subjects on ART induced a comparable response to two immunisations in ART naive subjects. There is considerable evidence to suggest that IFN-γ is necessary for protective immunity against *M. tb*, but it may not be sufficient [38]–[40]. Limited blood volumes in this study meant it was not possible to evaluate in more detail the cellular immune response induced by vaccination. Further studies are needed to evaluate the effects of 1 and 2 immunisations on other aspects of cellular immunity, including the more functional mycobacterial growth inhibition assays [41]. Furthermore, given the promising safety and immunogenicity data from this study, evaluation of the protective efficacy of this vaccine in HIV- infected subjects is merited. A phase IIb proof-of-concept efficacy trial is ongoing in HIV-infected subjects in South Africa and Senegal which will address this issue (clinicaltrials.gov identifier NCT01151189).

## Supporting Information

Checklist S1 CONSORT Checklist.
(DOC)

Protocol S1 Trial Protocol.
(PDF)
