# Correction: Two Doses of Candidate TB Vaccine MVA85A in Antiretroviral Therapy (ART) Naïve Subjects Gives Comparable Immunogenicity to One Dose in ART+ Subjects

**DOI:** 10.1371/annotation/67550546-cacf-4063-8d62-d165da14ca61

**Published:** 2013-10-15

**Authors:** Tandakha N. Dieye, Birahim P. NDiaye, Alle B. Dieng, Marema Fall, Nathaniel Brittain, Samantha Vermaak, Makhtar Camara, Halimatou Diop-Ndiaye, Ndeye Fatou Ngom-Gueye, Papa A. Diaw, Coumba Toure-Kane, Papa S. Sow, Souleymane Mboup, Helen McShane

The name of the fifth author was spelled incorrectly. 

The correct name is: Nathaniel Brittain. 

The correct citation is: Dieye TN, NDiaye BP, Dieng AB, Fall M, Brittain N, et al. (2013) Two Doses of Candidate TB Vaccine MVA85A in Antiretroviral Therapy (ART) Naïve Subjects Gives Comparable Immunogenicity to One Dose in ART+ Subjects. PLoS ONE 8(6): e67177. doi:10.1371/journal.pone.0067177. 

