# Supplementary material for: Two Doses of Candidate TB Vaccine MVA85A in Antiretroviral Therapy (ART) Naïve Subjects Gives Comparable Immunogenicity to One Dose in ART+ Subjects
Source: PLoS One. 2013 Jun 28;8(6):e67177. doi: 10.1371/journal.pone.0067177 (PMC3696007; doi:10.1371/journal.pone.0067177)
Supplement: Protocol S1 — Trial Protocol. (PDF) [file pone.0067177.s002.pdf]

UNIVERSITY OF OXFORD

CLINICAL TRIAL PROTOCOL

Study Reference: TB019

Oxford Tropical Research Ethics Committee Reference: 04/08
Comité National d'Ethique de la Recherche en Santé Reference: SEN 05/09

Title: A Phase I study evaluating the safety and immunogenicity of a new TB vaccine, MVA85A, in healthy volunteers who are infected with HIV

Sponsor: University of Oxford

Principal Investigator: Prof Souleymane Mboup

Chief Investigator: Dr Helen McShane

Local Safety Monitor: Prof Papa Salif Sow

Version Number: 2.1

11 June 2009

Amendments

Changes in bold and underlined.

Section of protocol	Previous text (Version 2.0)	New text (Version 2.1)
**Cover page**	**Conseil** National de Recherche en Santé Référence: **SEN 03/08**	**Comité National d'Ethique de la Recherche en Santé Référence: SEN 05/09**
**Protocol Summary Description of Agent /Intervention**: (Page viii)	MVA85A; 1 x 10^8 pfu, intradermally given at 0 and ~6-8 months	MVA85A; 1 x 10^8 pfu, intradermally given at 0 and **~6-12 months**
**Rationale** (Page 5)	We would therefore like to boost subjects in this study in both the ARV and no ARV group with a second MVA85A immunization, administered ~6-8 months after primary immunization. Subjects already recruited into the first (no ARV) arm will be contacted, informed of the results so far and asked if they would be happy to receive a second immunization. They are clearly under no obligation to do so and this will be clearly explained to them. Subjects in the second (ARV) arm of this study will be consented to receive two immunisations with MVA85A, with ~6-8 months between the two doses. It is thought that by boosting ~6-8 months after the first vaccination that the anti-vector immunity will have waned and should not impact the potential boosting by the second MVA85A vaccination.	We would therefore like to boost subjects in this study in both the ARV and no ARV group with a second MVA85A immunization, administered **~6-12 months** after primary immunization. Subjects already recruited into the first (no ARV) arm will be contacted, informed of the results so far and asked if they would be happy to receive a second immunization. They are clearly under no obligation to do so and this will be clearly explained to them. Subjects in the second (ARV) arm of this study will be consented to receive two immunisations with MVA85A, with **~6-12 months** between the two doses. It is thought that by boosting **~6-12 months** after the first vaccination that the anti-vector immunity will have waned and should not impact the potential boosting by the second MVA85A vaccination.
**5 STUDY ENROLLMENT AND SCHEDULE OF FOLLOW-UP VISITS**<br><br>***Tables 2 and 3*** *(Pages 13 and 14)*	Not applicable	- For table 2: the visit window of the additional visit 12.1^ has been extended to 350 days.<br>- For table 3:<br>**- a visit 12.1^ has been added with the following foot note " ^ FBC, Biochemistry, CD4 and VL will be repeated for exclusion criteria evaluation if a subject has been for their 6 month visit but was not vaccinated because the 6 month visit was prior to the second**

		**<u>vaccination amendment".</u>**<br>- The blood volumes in this table have been accordingly updated.
**Study visit**<br><br>**Booster vaccination**<br><br>(Page 16)	• For Group 1, if Day 168 visit is before Protocol 2.0 amendment, the second vaccination will be given between days 168-240 (visit 12.1).<br><br>• For Group 2, the second vaccination will be given at Day 168 (+/- 14 days)	• For Group 1, if Day 168 visit is before Protocol 2.0 amendment, the second vaccination will be given between days **<u>168-350</u>** (visit 12.1). H**<u>owever, before the subject receives the second vaccination, all inclusion and exclusion criteria except number 5 (Prior receipt of a recombinant MVA or Fowlpox vaccine) will be reviewed to check if the subject is still eligible to receive the vaccine. At this additional visit 20 ml of blood will be collected to perform FBC, Biochemistry, CD4 and VL tests. Urinalysis, to exclude glycosuria and significant proteinuria will be performed. If any exclusion criterion are met except number 5, the subject will not be revaccinated.</u>**<br>• For Group 2, the second vaccination will be given at Day 168 (+/- 14 days).<br>• **<u>A photograph of the vaccination site will be taken after revaccination. The subject will only be identifiable by a unique study number.</u>**
**Study visits**<br>(Pages 15, 16 and 17)	• The injection site will be inspected and the largest diameter of any hardness or redness present, as well as the degree of pain reported for the time of the visit will be documented into the CRF **<u>and diary card</u>**.	• The injection site will be inspected and the largest diameter of any hardness or redness present, as well as the degree of pain reported for the time of the visit will be documented into the CRF.

Table of Contents

Page
Title Page……i
Amendments……ii
Table of Contents……iii
Statement of Compliance……v
Signature Page……vi
List of Abbreviations……vii
Protocol Summary……viii
Key Roles……ix
Individuals……x
1 Background Information and Scientific Rationale……1
The need for new vaccine against tuberculosis……1
The development of a novel vaccination strategy against TB……1
The inclusion of antigen 85A in a heterologous prime-boost strategy……2
Using recombinant poxvirus vectors with BCG in a heterologous prime-boost strategy against TB……2
Recombinant modified vaccinia virus Ankara as a vector……2
Clinical studies using MVA85A……3
Table 1: Summary of clinical trials with MVA85A……4
Rationale……5
Potential Risks and Benefits……6
Potential risks……6
Known Potential Benefits……7
2 Objectives……8
3 Study Design……9
Sample size……9
4 Study Population……10
Inclusion Criteria……10
Exclusion Criteria……10
Recruitment Procedures……11
Screening visit……11
5 Study enrolment and schedule of follow-up visits……13
Table 2: Illustration of sequential events……13
Table 3: Temporal course of blood investigations……14
Definitions……14
Study visits……15
Study Day 0 (Day of vaccination)……15
Contraindications to vaccinations……15
Days 1–6 post vaccination……15
Day 7 post vaccination……16
Subsequent follow-up visits……16

Booster vaccination……16
Days 1-6 post second vaccination……16
Day 7 post second vaccination……17
Subsequent follow-up visits……17
6 Study Intervention/Investigational Product……18
Study Product Acquisition……18
Formulation, Packaging and Labelling……18
Product Storage and Stability……18
Preparation, Administration and Dosage of Study Intervention/Investigational Product……18
7 Assessment of Scientific Objectives (e.g., Safety or Immunogenicity or Efficacy)……19
Specification of the Appropriate Outcome Measures……19
Primary Outcome Measures……19
Secondary……19
8 Assessment of Safety……20
Definitions……20
Adverse Event……20
Serious Adverse Event (SAE)……20
Adverse Events Assessment……21
Serious Adverse Event (SAE) Reporting……22
Adverse Event Monitoring……22
SUSAR……22
Local Safety Monitor……22
Methods and Timing for Assessing, Recording, and Analyzing Safety Parameters……22
Reporting of Pregnancy……23
Procedures to be Followed in the Event of Abnormal Laboratory Test Values or Abnormal Clinical Findings……23
Halting Rules……23
9 Clinical Monitoring Structure……24
Site Monitoring Plan……24
Set-up Visit……24
Follow-up Visits……24
Close-out Visit……25
Audits and Inspections……25
10 Statistical Considerations……26
Determination of the Sample Size……26
Statistical Methods……26
Data Management……26
11 Source documents and Access to Source Data/Documents……27
12 Quality Control and Quality Assurance……28
13 Ethics/Protection of Human Subjects……29
Informed Consent Process……29
Subject Confidentiality……29
14 Data Handling and Record Keeping……30

Study Records Retention..30
Protocol Deviations 30
15 References……………………..…………………………………………………...……31

Statement of Compliance

This trial will be conducted in compliance with the protocol, International Conference on Harmonisation Good Clinical Practice E6 (ICH-GCP) and the EU Clinical Trials Directive (2001/20/EEC).

SIGNATURE PAGE

The signature below constitutes the approval of this protocol and the attachments, and provides the necessary assurances that this trial will be conducted according to all stipulations of the protocol, including all statements regarding confidentiality, and according to local legal and regulatory requirements and applicable UK and Senegal regulations and ICH guidelines.

Principal Investigator:

Signed: ____________________________________ Date: ________________

Name *Prof Souleymane Mboup*
Title *PharmD, PhD*

Chief Investigator:

Signed: ____________________________________ Date: ________________

Name *Dr Helen McShane*
Title *Wellcome Trust Senior Clinical Fellow*

Lead Clinician:

Signed: ____________________________________ Date: ________________

Name *Dr Birahim Pierre Ndiaye*
Title *MD, MPH*

List of Abbreviations

AE	Adverse event
ARV	Anti-retroviral therapy
ALT	Alanine aminotransferase
CHU	Centre Hospitalier Universitaire le Dantec
CMI	Cell-mediated immunity
CNRS	Conseil national de recherche en santé
CRF	Case Report Form
CRN	Clinical Research Nurse
EBV-B	Epstein-Barr virus transformed B cells
ELISA	Enzyme-linked immunosorbant assay
ELISPOT	Enzyme-linked immunospot
FBC	Full blood count
γIFN	Gamma interferon
HBV	Hepatitis B virus
HIV	Human immunodeficiency virus
HLA	Human leukocyte antigen
LFT	Liver function test
LSM	Local safety monitor
M.tb	*Mycobacterium tuberculosis*
MHRA	Medicines and Healthcare products Regulatory Agency
MVA85A	recombinant modified vaccinia virus Ankara expressing antigen 85A
NICE	National Institute of Clinical Excellence
OXTREC	Oxford Tropical Research Ethics Committee
PBMC	Peripheral blood mononuclear cells SAE Serious adverse event
SoAE	Solicited Adverse event
SUSAR	Suspected unexpected serious adverse reactions

Protocol Summary

Title: A Phase I study evaluating the safety and immunogenicity of a new TB vaccine, MVA85A, in asymptomatic volunteers who are infected with HIV.

Phase: I

Population: 24 healthy adult volunteers, aged 18–50, who are infected with HIV – 12 who are and 12 who are not on antiretroviral therapy

Number of Sites: 1: Centre Hospitalier Universitaire le Dantec, Dakar, Senegal
2 : Centre Régional de Recherche Clinique et de Formation (CRRCF), Centre Hospitalier Universitaire de Fann.

Study Duration: 24 months

Description of Agent /Intervention: MVA85A; 1 x 10^8 pfu, intradermally given at 0 and ~6-12 months

Objectives: Primary:

Safety of MVA85A in subjects who are infected with HIV

Secondary:

Immune responses induced by MVA85A in subjects who are infected with HIV

Immune responses induced by second MVA85A vaccination

To compare the immune response between subjects who are and who are not on ARVs after vaccination with MVA85A

Description of Study Design:

This is an open Phase I study of a candidate TB vaccine, MVA85A, in healthy subjects who are infected with HIV.

Key Roles

For questions regarding this protocol, contact:

Prof Souleymane Mboup and Dr Birahim Pierre Ndiaye
Laboratoire de Bacteriologie Virologie
Universite Cheikh Anta DIOP
CHU Le Dantec
BP 7325
Dakar
Senegal
Phone: (+221) 8216420 / 8225919
Fax: (+221) 8216442
Email: virus@orange.sn
mboup@eci.harvard.edu
mboup@rarslbv.org
ndaoct@ird.sn

Dr Helen McShane,
Centre for Clinical Vaccinology and Tropical Medicine,
Churchill Hospital,
Oxford
OX3 7LJ
Phone: (+44) 01865 857417
Email: helen.mcshane@ndm.ox.ac.uk

Institutions:

Sponsor:	University of Oxford, Wellington Square, Oxford OX1 2JD
Monitor:	This study will be independently monitored by contract monitors.
Study sites:	Centre Hospitalier Universitaire Le Dantec, BP 7325, Dakar, Senegal
Clinical laboratory:	Routine laboratory investigations to be carried out at Centre Hospitalier Universitaire Le Dantec and at the BIO24 laboratory.

Individuals:

Sponsor:
University of Oxford.

Investigators:
Dr Helen McShane, Wellcome Trust Senior Clinical Fellow, CCVTM, Churchill Hospital, Oxford, OX3 7LJ, UK. Phone: 01865 857417 helen.mcshane@ndm.ox.ac.uk

Prof Souleymane Mboup, Laboratoire de Bacteriologie Virologie, Universite Cheikh Anta DIOP, CHU Le Dantec, BP 7325, Dakar, Senegal. Phone: (+221) 33 8216420 / 33 8225919 virus@orange.sn, mboup@eci.harvard.edu, mboup@rarslbv.org

Lead Clinician:
Dr Birahim Pierre Ndiaye, Laboratoire de Bacteriologie Virologie, Universite Cheikh Anta DIOP, CHU Le Dantec, BP 7325, Dakar, Senegal. Phone: (+221) 338 22 59 19 / 77 638 99 08 pbsidya@yahoo.com

Trial Clinician :
Dr Alle Baba Dieng, Centre Régional de Recherche Clinique et de Formation, Centre Hospitalier Universitaire de Fann. BP 5035, Dakar, Senegal. Phone : +221 33 869 81 88 Mobile: +221 77 552 00 14. Fax : +221 33 864 78 53. allebabad@yahoo.fr

Immunologists:
Dr Tandakha Dieye, PharmD, PhD : Laboratoire de Bacteriologie Virologie, Universite Cheikh Anta DIOP, CHU Le Dantec, BP 7325, Dakar, Senegal. Phone: (+221) 33 8216420 / 33 8225919, GSM: +221 77 657 70 54 tndieye@yahoo.co.uk , dieye@cnts-dakar.sn

Dr Farba Karam, PharmD : Laboratoire de Bacteriologie Virologie, Universite Cheikh Anta DIOP, CHU Le Dantec, BP 7325, Dakar, Senegal. Phone: (+221) 338216420 / 338225919. GSM: +221 77 531 87 38 karamfarba@hotmail.com

Nurse:
Aliou Sene, Centre Régional de Recherche Clinique et de Formation, Centre Hospitalier Universitaire de Fann. BP 5035, Dakar, Senegal. Phone : +221 33 869 81 88

Project Manager:
Dr Alison Lawrie, CCVTM, Churchill Hospital, Oxford OX3 7LJ, UK Phone: 01865 857382. alison.lawrie@ndm.ox.ac.uk

Programme Coordinator:
Dr Nathaniel Brittain, CCVTM, Churchill Hospital, Oxford OX3 7LJ, UK Phone: 01865 857428. nathaniel.brittain@ndm.ox.ac.uk

Local Safety Monitor:
Prof Papa Salif Sow, Université Cheikh Anta DIOP de Dakar, Service des Maladies Infectieuses Ibrahima DIOP Mar, CHNU de Fann, BP 5035, Dakar, Senegal Phone: (221) 33 869 18 81. Fax: (221) 33 864 78 53. salifsow@sentoo.sn

Local Administrator:
Mme Ndèye Astou Fall Cissé, Laboratoire de Bactériologie-Virologie, Hopital Le Dantec, BP 7318 Dakar, Senegal Phone: 221 33 842.92.33, Fax : (221) 33 842.92.34
gesrars@orange.sn

Local Database manager/Administrator :
Ousmane Diouf. Laboratoire de Bactériologie-Virologie, Hopital Le Dantec, BP 7318 Dakar, Senegal Phone: 221 33 842.92.33, Fax : (221) 33 842.92.34. odiouf@gmail.com

1 BACKGROUND INFORMATION AND SCIENTIFIC RATIONALE

The need for a new vaccine against tuberculosis

Tuberculosis (TB) is one of the leading causes of death from a single infectious agent and kills about three million people annually. One third of the world's population is latently infected with *Mycobacterium tuberculosis* (*M.tb*) [1]. These individuals have a 10% lifetime risk of developing active TB disease, or 10% annual risk if they become immunosuppressed [2]. If untreated, the five year mortality from TB disease is high. The highest numbers of cases are in Africa and South East Asia and the prevalence of TB is increasing worldwide, largely due to the increasing prevalence of HIV [3] [4] [5]. The only available vaccine, *M. bovis* BCG, is largely ineffective at protecting against adult pulmonary disease [6], but does protect against disseminated TB and tuberculous meningitis in children [7] [8]. Modelling studies of the TB epidemic suggest that 50 million people could die in the next 20 years, even if current control measures are aggressively implemented [9]. It is widely agreed that a new, more effective tuberculosis vaccine is a major global public health priority and could save millions of lives [10]. The WHO Stop TB partnership describes the development of a new, effective tuberculosis vaccine as 'an essential component of any strategy to eliminate TB by 2050' [11] [12].

The development of a novel vaccination strategy against TB

M.tb is an intracellular organism and protective immunity is dependant upon an intact cellular immune system. Both CD4 and CD8 Th1-type cellular responses are important for protection [13]. A new TB vaccine would need to induce strong cellular immune responses. Several antigen delivery systems are able to induce T-cells, such as DNA vaccines, recombinant viral vectors and protein/adjuvant combinations. However, when used alone, each of these systems can only induce low-level responses. Heterologous prime-boost immunisation strategies involve immunization with two different vaccines, each expressing the same antigen, several weeks apart. In disease models of malaria, TB and HIV, these induce higher levels of antigen specific CD4+ and CD8+ T cells than homologous boosting with the same vector [14] [15] [16]. Given the protective efficacy of BCG in childhood, ideally BCG would be the priming immunisation in such a prime-boost strategy. BCG is administered at birth in developing countries which are endemic for TB. Therefore, an immunisation strategy that includes BCG is also attractive because the populations in which this vaccine candidate will need to be tested will already have been immunised with BCG. We are aiming to develop an immunization strategy against TB, which retains the effectiveness of BCG in childhood, whilst improving protection against pulmonary disease in adults. A boosting vaccination could be given in infancy to boost BCG, or in later life to boost immunity induced by exposure to tuberculosis or environmental mycobacteria.

The inclusion of antigen 85A in a heterologous prime-boost strategy

The antigen that is used in a prime-boost strategy, where BCG is the priming immunization, needs to be present in all strains of BCG. Antigen 85A forms part of the antigen 85 complex (A, B and C), which constitutes a major portion of the secreted proteins of both *M. tuberculosis* and BCG. It is one of the most abundant proteins in short term culture filtrate of *M.tb*, constituting 15% [17]. It is highly conserved amongst all mycobacterial species, is present in all strains of BCG [18] and is a major target of BCG-induced immune responses [19]. Antigen 85A has mycolyltransferase and fibronectin-binding activity in the final stages of mycobacterial cell wall assembly and has been shown to be essential, making it an ideal target [20]. When used as a DNA vaccine, antigen 85A is protective in mice [21], and induces both CD4+ and CD8+ cells [22]. There is also evidence that antigen 85A is an immunodominant antigen in humans. CD4+ T cell epitopes have been identified in patients infected with TB [18] and HLA-A2 restricted CD8+ T-cells have been found in a high proportion of BCG-immunised individuals [23].

Using recombinant poxvirus vectors with BCG in a heterologous prime-boost strategy against TB

Recombinant viral vectors, such as poxviruses and adenoviruses, are a particularly effective way of boosting strong T cell responses when used in heterologous prime-boost strategies. The advantage of using a recombinant viral vector as a new TB vaccine is that recombinant technology allows for greater production of immunodominant antigens, which could result in a stronger and more effective immune response against tuberculosis. We have developed two recombinant poxvirus vectors that each encode a sequence from antigen 85A, modified vaccinia virus Ankara (MVA) and fowlpox virus 9 (FP). These are non-replicating strains, making them attractive in terms of safety. Furthermore, it has been shown that non-replicating strains can be more immunogenic than traditional replicating strains. In the murine model of TB, using BCG as the priming immunisation and then boosting with recombinant MVA encoding 85A (MVA85A) induces higher levels of both antigen specific IFN-γ secreting CD4+ and CD8+ T cells and significantly greater levels of protection against aerosol challenge than after BCG alone [24]. In rhesus macaques, BCG prime, with MVA boost is more immunogenic than any of the vaccines alone, and is more protective than BCG alone (Verrek et al, unpublished data). This regime has now been further evaluated in the more sensitive guinea pig aerosol challenge model with very encouraging results.

Recombinant modified vaccinia virus Ankara as a vector

The successful worldwide eradication of smallpox via vaccination with live vaccinia virus highlighted vaccinia as a candidate virus for recombinant use. MVA is a highly attenuated strain of vaccinia virus that is unable to replicate efficiently in human cell lines and most mammalian cells [25]. MVA underwent multiple, fully characterised deletions during more than 570 passages through chicken embryo fibroblast cells [26] including deletions in host range genes and genes encoding cytokine receptors. Viral replication is blocked at a late stage of virion assembly, so, importantly, viral and recombinant protein synthesis is unimpaired [27]. This means that MVA is an efficient single round expression vector, incapable of causing infection in mammals. Replication-deficient recombinant MVA has been seen as an exceptionally safe viral vector. It has been administered to more than 120,000 vaccinees as part of the smallpox eradication programme, with an excellent safety record, despite the deliberate vaccination of high risk groups [26]. This safety in man is consistent with the avirulence of MVA in animal models, where recombinant MVAs have also been shown to be protectively immunogenic as vaccines against viral diseases and cancer. Importantly for a

tuberculosis vaccine, recombinant MVAs expressing HIV antigens have been shown to be safe and immunogenic in HIV-infected subjects [28-30]. There is now safety data from many recombinant MVAs expressing HIV, malaria and melanoma antigens that are currently in phase I/II trials in both the UK and Africa [28-35].

Clinical studies using MVA85A

MVA85A has been administered (at a dose of 5 x 10^7pfu) to 57 healthy volunteers in the UK, 21 healthy adults and 8 healthy infants in The Gambia and 24 adults and 8 adolescents in South Africa, with no vaccine-related serious adverse events (table one). In addition to this, MVA85A low dose (1x10^7 pfu) and high dose (1x10^8 pfu) have each been given to 12 volunteers with no vaccine-related serious adverse events. All volunteers have temporary local redness with, typically, a 5mm central red area with a paler pink surrounding area that ranges in size from about 1-7mm in diameter and peaks at 48 hours post vaccination. At seven days post vaccination, generally only the central red area remains. This fades over the next few weeks and is not usually apparent at 2 months after vaccination.

There is a theoretical risk of a Koch reaction i.e. the development of immunopathology due to the stimulation of an exaggerated immune response. Boosting immunizations in individuals who have some pre-existing mycobacterial immunity may stimulate the immune response to such an extent that a Koch reaction develops. However, mouse data suggests that these reactions correlate with high mycobacterial load and are unlikely in healthy individuals, even if latently infected [36]. Our initial studies were performed in healthy volunteers with no evidence of pre-existing mycobacterial immunity, as assessed by Heaf testing and cellular immune responses to *M.tb* peptides, (PPD, ESAT 6 and CFP10), and who had not received BCG vaccination. We have now completed studies in the UK vaccinating volunteers previously vaccinated with BCG [37] and in The Gambia. These volunteers were excluded if their Heaf test was greater than grade II. A further study, involving vaccination of 12 volunteers who were latently infected with TB, is nearing completion and to date has had no vaccine-related serious adverse events. Importantly, in this study, the side effect and immunogenicity profile is the same as in previous studies and there have been no clinical, radiological or immunological signs of a Koch reaction. Phase II studies in 24 healthy *M.tb* infected adults and 12 adolescents commenced in Cape Town in August 2005 and the adult arm of this trial has now completed enrolment. Recruitment into the adolescent arm is ongoing.

Table 1: Summary of clinical trials with MVA85A

**Country**	**Volunteer Group**	**Dose of MVA85A**	**Number of volunteers**	**Status**
UK	BCG naïve and Heaf grade 0	5×10^7 pfu x 2 doses	14	Completed
UK	BCG-prime and MVA85A boost (1 month interval)	5×10^7 pfu	10	Completed
UK	BCG-prime and MVA85A boost (24 month interval)	5×10^7 pfu	5	Completed
UK	BCG-prime and MVA85A boost (10-20 year interval)	5×10^7 pfu	12	Completed
UK	BCG-prime and MVA85A dose-optimization	1×10^7 pfu 1×10^8 pfu	24 (12 each dose)	Completed
Gambia	BCG naïve and Heaf grade 0	5×10^7 pfu x 2 doses	11	Completed
Gambia	BCG-prime (birth) and MVA85A-boost	5×10^7 pfu	10	Completed
South Africa	BCG naïve or BCG-prime MVA85A-boost	5×10^7 pfu	24 (12 per group)	Enrolment complete
UK	Latent infection	5×10^7 pfu	12	Completed
The Gambia	BCG vaccinated infants	2.5 and 5×10^7 pfu	350	Recruiting
South Africa	Healthy BCG-vaccinated adolescents	5×10^7 pfu	12	Enrolment complete
UK	Asymptomatic HIV+	5×10^7 and 1×10^8 pfu	20	Recruiting
South Africa	HIV+/TB-, HIV-/TB+ or HIV+/TB+	5×10^7 pfu	36	Recruiting

Rationale

This study is designed to evaluate the safety of MVA85A in healthy volunteers in Senegal who are infected with HIV. In phase I studies, a single vaccination with MVA85A, when administered at a dose of 5 x 10^7pfu intradermally, has been shown to be safe in both mycobacterially naïve individuals, those previously vaccinated with BCG and latently infected individuals. We will use 1 x 10^8 pfu MVA85A intradermally in this study. A trial in BCG vaccinated subjects showed that the higher dose (1 x 10^8 pfu MVA85A) induced a significantly higher immune response but did not have a higher AE profile. In addition, because of a variable immune response, the trial in HIV positive subjects in the UK is split into two groups, the first getting 5 x 10^7 pfu and the second getting 1 x 10^8 pfu MVA85A. It has, therefore, been decided to use the higher dose in order to maximise the immune response whilst maintaining a good safety profile.

However, the T cell immunogenicity of MVA85A in HIV infected subjects who are not on ARV treatment is reduced compared to previous trials in HIV negative subjects.
There are two potential interventions we can do to improve the immunogenicity in this important target population where TB incidence is very high. First, it is possible that the immune response to MVA85A would be better in healthy, asymptomatic people who have a CD4 count of more than 300, but who have had their immune response reconstituted by ARV therapy. Therefore the rationale for adding a second arm to this study is to investigate the immunogenicity in a group of HIV infected subjects who are stable on ARV treatment.
It is important to determine which subjects have the best immune response to this vaccine, in order to select this group for inclusion in a proof-of-concept efficacy trial; this would maximize the prospects of demonstrating efficacy in this important target population.

The second possible way to enhance the immunogenicity in this group is to include a second MVA85A booster vaccine. In the first trial we conducted with MVA85A, we administered two doses of MVA85A, three weeks apart [37]. In this study we saw no impact of the second MVA85A immunization, and at such a short interval between doses, this is likely to be due to anti-vector immunity. Other studies using a recombinant MVA expressing malaria antigens have shown that boosting with the same vaccine one year after first dose is highly effective [31]. We would therefore like to boost subjects in this study in both the ARV and no ARV group with a second MVA85A immunization, administered ~6-12 months after primary immunization. Subjects already recruited into the first (no ARV) arm will be contacted, informed of the results so far and asked if they would be happy to receive a second immunization. They are clearly under no obligation to do so and this will be clearly explained to them. Subjects in the second (ARV) arm of this study will be consented to receive two immunisations with MVA85A, with ~6-12 months between the two doses. It is thought that by boosting ~6-12 months after the first vaccination that the anti-vector immunity will have waned and should not impact the potential boosting by the second MVA85A vaccination.

Potential Risks and Benefits

Potential risks

The general risks to participants in this Phase I study are associated with phlebotomy and with vaccination. The volume of blood drawn over the study period (652.5 +/- 20ml) should not compromise these otherwise healthy subjects. As vaccine-related side effects are believed to be related more to the vector used than the specific insert, it is expected that MVA85A will have a similar side effect profile to recombinant pox viruses encoding other antigens.

Potential risks include the following:

Local reactions: Mild tenderness, bruising, or fainting may result from venepuncture. An inflammatory reaction as manifested by redness, swelling, scaling and/or tenderness may occur at the site of vaccine injection. In previous studies using the vaccines MVA85A these local reactions have spontaneously resolved within weeks.

Systemic Reactions: Systemic reactions to immunization with vaccines using MVA as a carrier that could potentially occur include a flu-like illness with low-grade fever, chills and malaise. As with any other vaccine, temporary ascending paralysis, the Guillain-Barré syndrome (GBS) or immune mediated reactions that can lead to organ damage may occur. For influenza vaccines an excess of approximately 1 GBS case per million persons immunized has been observed. No cases were observed in people under 45 years of age. However, this has never been seen with these vaccines or vaccines containing any of their components. As with any vaccine, serious allergic reactions may occur.

Allergic Reactions and Anaphylaxis: As with any vaccine, allergic reactions are possible.

TB specific risks: There is a very small risk of a Koch reaction occurring when subjects latently infected with M.tb are vaccinated with MVA85A. All subjects will be closely monitored and followed up clinically. All volunteers found to be latently infected with M.tb will be offered treatment at the end of the trial, in accordance with the latest NICE guidelines (6 months of isoniazid).

HIV specific risks: There is a hypothetical risk of any vaccine causing a rise in viral load in HIV positive individuals. However in individuals established on ARVs this has not been seen with MVA vaccines [30]. A small number of HAART naïve individuals in Africa have also been vaccinated with a recombinant MVA with no significant or sustained rise in viral load or fall in CD4 count [32]. Volunteers will be monitored clinically together with regular monitoring of CD4 counts and viral loads. However, observed changes in CD4/viral load will have to be carefully interpreted in the context of a well-described natural fluctuation in these parameters. Any significant changes in these will be communicated to the volunteer's lead HIV physician forthwith and BHIVA guidelines (BHIVA treatment guidelines 2005) will be followed to determine the need to commence ARVs.

Risks associated with radiation exposure:
A chest-x ray will be performed if there is not an available film that has been performed in the last 3 months. The maximum radiation dose for one chest x-ray is 0.04 mSv, which is equivalent to 5 days of natural background radiation.

Known Potential Benefits:

Volunteers will not benefit directly from participation in this study. However, it is hoped that the information gained from this study will contribute to the development of a safe and effective TB vaccine for HIV negative and positive individuals. The only benefits for participants would be information about their general health status.

2 OBJECTIVES

Primary:

To assess the safety of two intradermal injections of 1 x 10^8 pfu MVA85A, when administered to healthy subjects who are infected with HIV.

Secondary:

To assess the immune responses induced by two vaccinations with MVA85A in healthy subjects who are infected with HIV, including the boosting affect of second MVA85A vaccination

To compare the immune response between subjects who are and who are not on ARVs after vaccination with MVA85A

Endpoints:

The specific endpoints for safety and reactogenicity will be actively and passively collected data on adverse events (AEs). The specific endpoints for immunogenicity will be markers of cell-mediated immunity.

3 STUDY DESIGN

This is an open label Phase I safety study of two intradermal injections of 1 x 10^8 pfu MVA85A, when administered to healthy subjects who are infected with HIV.

Sample size:

This is an observational and descriptive safety study, 24 subjects will be recruited from HIV clinics and vaccinated with MVA85A. Half of the subjects will not have started on ARVs and half will be stable on ARVs. This sample size should allow determination of the magnitude of the outcome measures, especially of serious and severe adverse events, rather than aiming to obtain statistical significance.

End of trial:

The trial ends when the last volunteer has completed their last (twelve month) follow-up visit.

4 STUDY POPULATION

All subjects must meet all of the inclusion criteria to participate in this study.

Inclusion Criteria

- Healthy adults aged 18 to 50 years
- Resident in or near Dakar for the duration of the study
- Willingness to allow the investigators to discuss the volunteer's medical history with the volunteer's HIV lead physician
- Willing to use effective contraception throughout duration of study (if female)
- Written informed consent

Group 1 (HIV infected not on ARV):

- HIV antibody positive; diagnosed at least 6 months previously
- CD4 count >300 and CD4 count nadir 300
- HIV viral load not >100,000 copies per millilitre

Group 2 (HIV infected on ARV):

- HIV antibody positive; diagnosed at least 12 months previously
- Two CD4 counts prior to enrolment >300; and CD4 count nadir 100
- Stable on same ARV regimen for at least 12 months
- Undetectable HIV viral load for at least 12 months

Exclusion Criteria

- Any clinically significant abnormal finding on screening biochemistry or haematology blood tests or on urinalysis
- Previous history of TB disease and/or treatment
- Any AIDS defining illness
- CXR showing TB or evidence of other active infection
- Prior receipt of a recombinant MVA or Fowlpox vaccine
- Use of any investigational or non-registered drug, live vaccine or medical device other than the study vaccine within 30 days preceding dosing of study vaccine, or planned use during the study period
- Administration of chronic (defined as more than 14 days) immunosuppressive drugs or other immune modifying drugs within six months of vaccination. (For corticosteroids, this will mean prednisolone, or equivalent, ≥ 0.5 mg/kg/day. Inhaled and topical steroids are allowed.)
- History of allergic disease or reactions likely to be exacerbated by any component of the vaccine, e.g. egg products
- Presence of any underlying disease that compromises the diagnosis and evaluation of response to the vaccine (including evidence of cardiovascular disease, history of cancer (except basal cell carcinoma of the skin and cervical carcinoma in situ), history of insulin requiring diabetes mellitus, any ongoing chronic illness requiring ongoing specialist supervision (e.g., gastrointestinal), and chronic or active neurological disease)

- History of ≥ 2 hospitalisations for invasive bacterial infections (pneumonia, meningitis)
- Suspected or known current drug and/or alcohol abuse
- Seropositive for hepatitis B surface antigen (HBsAg) and/ or hepatitis C (antibodies to HCV)
- Evidence of serious psychiatric condition
- Any other on-going chronic illness requiring hospital specialist supervision
- Any confirmed or suspected immunosuppressive or immunodeficient condition, **other than** HIV infection, such as asplenia
- Evidence of hepatomegaly
- Administration of immunoglobulins and/or any blood products within the three months preceding the planned administration of the vaccine candidate
- Pregnant/lactating female and any female who is willing or intends to become pregnant during the study
- Any history of anaphylaxis in reaction to vaccination
- PI assessment of lack of willingness to participate and comply with all requirements of the protocol, or identification of any factor felt to significantly increase the participant's risk of suffering an adverse outcome

Group 1 (HIV infected not on ARV):

- Current CD4 count less than 300 or CD4 count nadir less than 300
- Any ARV therapy within the last six months

Group 2 (HIV infected on ARV):

- CD4 count less than 300 or CD4 count nadir less than 100
- Detectable HIV viral load at >75 copies/ml

Recruitment Procedures

Patients will be screened from the Fann Teaching Hospital Ambulatory Treatment Centre (CTA)

Screening visit

The screening will take place at the Centre Régional de Recherche Clinique et de Formation at the Fann Teaching hospital which is in front of the CTA (less than 100 metres). The information sheet will be made available to the volunteer at least 24 hours prior to the screening visit. The screening visit may take place up to 90 days prior to vaccination. At the screening visit, before screening is undertaken, the screening process will be reviewed in detail with each prospective volunteer and all questions about the screening process answered. Each potential subject will be fully informed so that they understand the following general principles:

- Participation in the study is entirely voluntary
- Refusal to participate involves no penalty or loss of medical benefits
- The subject may withdraw from the study at any time

- The subject is free to ask questions at any time to allow him or her to understand the purpose of the study and the procedures involved
- The study involves research of an investigational vaccine
- There is no direct benefit for participating

The aims of the study and all tests to be carried out will be explained by the investigator. Written informed consent will be obtained. Potential subjects will be informed that there may be leftover samples of their blood (after all testing for this study is completed), and that such samples may be stored up to a maximum of 15 years for possible future research (exploratory immunology assays). Subjects will be able to decide if they will permit such future use of any leftover samples. If a subject elects not to permit this, all of that subject's leftover samples will be discarded after the required period of storage to meet Good Clinical Practice (GCP) and regulatory requirements.

Events at screening:

- A baseline medical history and physical examination will be performed
- Inclusion and exclusion criteria will be checked using a tabulated format
- Vital signs will be checked
- Subjects will be counselled by one of the investigators for Hepatitis B and Hepatitis C testing
- Blood will be drawn for HIV, HBV and HCV testing, haematology, biochemistry, viral load and CD4 cell count, typing for HLA variants, other genetic variants that may be relevant to the immune response to vaccines, and exploratory immunological assays. A serum βHCG test will be done on women to test for pregnancy.
- Urinalysis, to exclude glycosuria and significant proteinuria will be performed
- A chest-x ray will be performed if there is not an available film that has been performed in the last 3 months.
- 1 week after screening, volunteers will attend to obtain their results.

All documents and procedures will be in French, having been translated and backtranslated from English. Wolof translators will be present when necessary.

5 STUDY ENROLLMENT AND SCHEDULE OF FOLLOW-UP VISITS

Subjects who match all inclusion and exclusion criteria and who are available for the follow up will be invited to take part after screening. Subjects will attend the Bacteriology and Virology Laboratory at Le Dantec Teaching Hospital for vaccination. All volunteers will be vaccinated with MVA85A and will not be randomised.

The following table illustrates the sequence of events from screening, through vaccination (day 0) and subsequent follow-up visits (explained in more detail in the text that follows):

Table 2: Illustration of sequential events (screening and subsequent follow-up):

Visit number	1	2	3–8	9	10	11	12	12.1^	13-18	19	20	21	22
**Day**	**Screening**	**0**	**1–6**	**7**	**28**	**84**	**168** (+/-14)	**168-350**	**1–6◊**	**7**	**28**	**84**	**168**
**Medical history**	√	(√)	(√)	(√)	(√)	(√)	(√)	(√)	(√)	(√)	(√)	(√)	(√)
**Physical examination**	√	(√)	(√)	(√)	(√)	(√)	(√)	(√)	(√)	(√)	(√)	(√)	(√)
**Vital signs°**	√	√	√	√	√	√	√	√	√	√	√	√	√
**Urinalysis**	√	√ †					√ †	√†					
**Chest x-ray#**	√												
**Venepuncture**	√∗	√∗		√∗	√∗	√∗	√∗	√∗		√∗	√∗	√∗	√∗
**Vaccination**		√					√	√					
**Injection site inspection**		√	√	√	√	√	√	√	√	√	√	√	√
**Adverse events documented**		√	√	√	√	√	√	√	√	√	√	√	√

(√) If considered necessary (emphasizing any acute complaints)
^ Visit 12.1 is only required if a subject has been for their 6 month visit but was not revaccinated because the 6 month visit was prior to the second vaccination amendment

° Temperature (^{O}C), blood pressure (mmHg), pulse rate (beats/minute)
† Women only for pregnancy test
# Performed if no available CXR within the last 3 months
∗ See table 3 for exact blood tests performed at each visit
◊ Note that the days from this point on are shown as days after the second vaccination

Table 3: Temporal course of blood investigations:

Test/Timepoint	Volume (ml)	Scr	0	7	28	84	168 (+/- 14)	12.1^ (168-350)	7◊	28	84	168	Total (ml)
FBC	4	**X**		**X**		**X**		**X**	**X**		**X**		20-24
BioChem	4	**X**		**X**		**X**		**X**	**X**		**X**		20-24
HBV/HCV/β-HCG	4	**X**											4
HIV	4	**X**											4
CD4	4	**X**	**X**	**X**	**X**	**X**	**X**	**X**	**X**	**X**	**X**	**X**	40-44
Viral Load	8	**X**	**X**	**X**	**X**	**X**	**X**	**X**	**X**	**X**	**X**	**X**	80-88
HLA	4				**X**								4
Research Bloods	20-30 (16-26 at screening)	**X**	**X**	**X**	**X**	**X**	**X**		**X**	**X**	**X**	**X**	216-326
TOTAL	–	44-54	32-42	40-50	36-46	40-50	32-42	0-20	40-50	32-42	40-50	32-42	364-484

◊ Note that the days from this point on are shown as days after the second vaccination
^ FBC, Biochemistry, CD4 and VL will be repeated for exclusion criteria evaluation if a subject has been for their 6 month visit but was not vaccinated because the 6 month visit was prior to the second vaccination amendment.

Definitions

The following definitions/clarifications outline the blood investigations that will be performed:
Haematology* – full blood count (FBC) including platelet count
Biochemistry* – urea, creatinine, albumin, ALT, alkaline phosphatase, potassium, sodium, total bilirubin
Diagnostic serology* – hepatitis B surface antigen, hepatitis B surface antibody, hepatitis B core antibody and hepatitis C serology (IgG)
Pregnancy test*– β-HCG (for women only)
Exploratory immunology** –Ex-vivo Elispot assays for interferon-gamma
Specific to HIV** – CD4 count, HIV serology and viral load.

* Assays will be carried out at BIO 24 Laboratory
** Assays will be carried out at CHU, Dakar, using standard procedures

Of the 20-30 ml blood taken for immunological assays, half will be required for immediate use, the other half will be stored as future research source.
In addition:

- Plasma and cells will be stored separately.
- Samples will be stored for up to 15 years.
- Plasma will be frozen down and stored for possible future use.

Study Visits:

Study Day 0 (Day of vaccination)

- An interim history will be taken and if considered necessary a physical examination emphasizing any acute complaints will be performed.
- A β-HCG urine test will be performed for female volunteers. Females will not undergo injection until the test is completed and reported as negative.
- Approximately 32-42 ml of blood will be collected for CD4 count, viral load and "exploratory immunology" tests.
- The vaccine candidate will be administered intradermally into the skin over the deltoid muscle of the appropriate arm. The injection site will be covered with a sterile dressing that will be removed at the one-hour observation.
- Vital signs will be checked at 30 minutes (+/- 5 mins) and 1hour (+/-10min) post injection.
- The injection site will be inspected at 1 hour (+/-10min) and the largest diameter of any hardness or redness as well as the degree of pain will be documented into the CRF. An assessment for possible systemic AE's will be carried out.
- It will be explained to the subject that there will be an active follow up for the first week, which will be at CRRCF-Fann by the trial nurse and/or clinician. The details of the visits (i.e. temperature, adverse event monitoring etc.) will also be described.
- A photograph of the vaccination site will be taken. The subject will only be identifiable by a unique study number.

Contraindications to Vaccination

The following adverse events constitute contraindications to administration of vaccine at that point in time; if any one of these adverse events occurs at the time scheduled for vaccination, the subject may be vaccinated at a later date, or withdrawn at the discretion of the investigator. The subject must be followed until resolution of the event as with any adverse event.

- Acute disease at the time of vaccination. (Acute disease is defined as the presence of a moderate or severe illness with or without fever.) All vaccines can be administered to persons with a minor illness such as diarrhoea, mild upper respiratory infection with or without low-grade febrile illness, i.e., temperature of <37.5°C).

- Temperature of ≥37.5°C at the time of vaccination

Days 1–6 post vaccination (+/- 0 day)

- An interim history will be taken and, if considered necessary a physical examination emphasizing any acute complaints will be performed. Vital signs will be checked and documented
- The injection site will be inspected and the largest diameter of any hardness or redness present, as well as the degree of pain reported for the time of the visit will be documented into the CRF.
- Solicited adverse events (local and general) that may have occurred since the vaccination will be documented in the CRF.

Day 7 (+ 1 day)

- An interim history will be taken and, if considered necessary a physical examination emphasizing any acute complaints will be performed. Vital signs will be checked and documented
- The injection site will be inspected and the largest diameter of any hardness or redness present, as well as the degree of pain reported for the time of the visit will be documented into the CRF.
- Solicited adverse events (local and general) that may have occurred since the vaccination will be documented in the CRF.
- Approximately 40-50 ml of blood will be collected for haematology, biochemistry, viral load, CD4 cell count and exploratory immunology.
- A photograph of the vaccination site will be taken. The subject will only be identifiable by a unique study number.

Subsequent follow-up visits

Volunteers will be reviewed at 28 (+/- 4 days), 84 (+/- 7 days) and 168 days (+/- 14 days) after vaccination.

- An interim history will be taken and, if considered necessary a physical examination emphasizing any acute complaints will be performed.
- Vital signs will be checked and documented
- The injection site will be inspected and the largest diameter of any hardness or redness present, as well as the degree of pain reported for the time of the visit will be documented into the CRF.
- Solicited adverse events (local and general) that may have occurred since the volunteer was seen on day 7 post vaccination will be documented in the CRF
- Approximately 32-42 ml of blood will be collected for exploratory immunology, viral load and CD4 cell count on day 28, 84 and 168. On day 84 only, haematology and biochemistry will also be performed; therefore, the blood volume will be 40-50 ml.
- On Day 28, HLA typing will be done; therefore, the total blood volume will be 36-46.

Booster vaccination:

- For Group 1, if Day 168 visit is before Protocol 2.0 amendment, the second vaccination will be given between days 168-350 (visit 12.1). However, before the subject receives the second vaccination, all inclusion and exclusion criteria except number 5 (Prior receipt of a recombinant MVA or Fowlpox vaccine) will be reviewed to check if the subject is still eligible to receive the vaccine. At this additional visit, 20 ml of blood will be collected to perform FBC, Biochemistry, CD4 and VL tests. Urinalysis, to exclude glycosuria and significant proteinuria will be performed. If any exclusion criterion are met except number 5, the subject will not be revaccinated.

- For Group 2, the second vaccination will be given at Day 168 (+/- 14 days)

- A photograph of the vaccination site will be taken after revaccination. The subject will only be identifiable by a unique study number.

Days 1–6 post second vaccination (+/- 0 day)

- An interim history will be taken and, if considered necessary a physical examination emphasizing any acute complaints will be performed. Vital signs will be checked and documented

- The injection site will be inspected and the largest diameter of any hardness or redness present, as well as the degree of pain reported for the time of the visit will be documented into the CRF.
- Solicited adverse events (local and general) that may have occurred since the vaccination will be documented in the CRF.

Day 7 (+/- 1 day) post second vaccination

- An interim history will be taken and, if considered necessary a physical examination emphasizing any acute complaints will be performed. Vital signs will be checked and documented
- The injection site will be inspected and the largest diameter of any hardness or redness present, as well as the degree of pain reported for the time of the visit will be documented into the CRF.
- Solicited adverse events (local and general) that may have occurred since the vaccination will be documented in the CRF.
- Approximately 40-50 ml of blood will be collected for haematology, biochemistry, viral load, CD4 cell count and exploratory immunology.
- A photograph of the vaccination site will be taken. The subject will only be identifiable by a unique study number.

Subsequent follow-up visits

Volunteers will be reviewed at 28 (+/- 4 days), 84 (+/- 7 days) and 168 days (+/- 14 days) after the second vaccination.

- An interim history will be taken and, if considered necessary a physical examination emphasizing any acute complaints will be performed.
- Vital signs will be checked and documented
- The injection site will be inspected and the largest diameter of any hardness or redness present, as well as the degree of pain reported for the time of the visit will be documented into the CRF.
- Solicited adverse events (local and general) that may have occurred since the volunteer was seen on day 7 post second vaccination will be documented in the CRF.
- Approximately 32-42 ml of blood will be collected for exploratory immunology, viral load and CD4 cell count on days 28, 84 and 168 after the second vaccination. On day 84 after the second vaccination only, haematology and biochemistry will also be performed; therefore, the blood volume will be 40-50 ml.

6 STUDY INTERVENTION / INVESTIGATIONAL PRODUCT

Study Product Acquisition

Formulation, Packaging and Labelling

MVA85A is manufactured to Good Manufacturing Practice conditions by Impfstoffwerk Dessau-Tornau (IDT), Germany, for Oxford University. It is supplied in glass vials. These vials are clearly labelled with MVA85A.

Product Storage and Stability

MVA85A will be stored at –80°C in a locked freezer at Laboratoire de Bactériologie-Virologie, CHU Le Dantec, Dakar.

Preparation, Administration and Dosage of Study Intervention/Investigational Product

The vaccine, MVA85A, is provided in vials of 200 microlitres volume at a concentration of 7.4 x 10^8 pfu/ml in 10mM Tris buffer. The dose of MVA85A to be used in this study will be 1 x 10^8 pfu (135µl), this same dose will be given twice. On both vaccination days, the vaccine will be allowed to thaw to room temperature and administered within 1 hour. A single vaccine vial per subject to be immunised will be used on both vaccination days. The vaccine will be administered intradermally over the deltoid region of one arm. The investigator will wear gloves and eye protection. Subjects will stay in the unit for 60 minutes (+/- 10 mins) after vaccination for observation. During administration of the vaccines, medicines and resuscitation equipment will be immediately available for the management of anaphylaxis.

In order to minimise dissemination of the recombinant vectored vaccine virus into the environment the inoculation sites will be covered with a dressing after immunisation. This should absorb any virus that may leak out through the needle track. The dressings will be removed from the injection sites at the end of the 60 minute observation period and will be disposed as GMO waste by autoclaving, in accordance with the relevant SOP and current standard practice.

7 ASSESSMENT OF SCIENTIFIC OBJECTIVES (E.G., SAFETY OR IMMUNOGENICITY OR EFFICACY)

Specification of the Appropriate Outcome Measures

Primary Outcome Measures:

To assess the safety of two intradermal injections of 1 x 10^8pfu MVA85A, when administered to healthy subjects who are infected with HIV. The specific endpoints for safety and reactogenicity will be actively and passively collected data on adverse events (AEs).

Secondary:

To assess the effect of vaccination with MVA85A in healthy subjects who are infected with HIV on the immune response to antigen 85A (the antigen in the vaccine) and to ESAT-6 and CFP-10 (markers of TB infection).
The effect of a booster vaccination at six months will also be assessed.
A comparison between the immune responses by HIV-infected people who are not and are on ARVs will also be done.

The specific endpoints for immunogenicity will be markers of cell-mediated immunity as outlined below:

- Ex-vivo Elispot assay for interferon gamma: (exploratory immunology)

In all previous TB vaccine trials this has been the main immunological readout. However, there are as yet no validated cellular immunological assays and no perfect correlate of protection has been identified for TB. There is therefore ongoing work in the laboratory to develop other cellular assays including flow cytometry tests.

In view of this, other immunological assays may include:

- ex-vivo elispot assays for interleukin-2 and tumour necrosis factor alpha
- cultured elispot
- T cell proliferation
- whole blood assay for interferon-gamma
- T cell depletion assays
- cytokine detection using ELISA/LUMINEX or
- intracellular staining and FACScan analysis will be performed at the discretion of the investigators. (This also includes gene expression studies.)
- any other immunological assays that are developed during the timeline of the trial

8 ASSESSMENT OF SAFETY

All adverse events (AE) occurring in participants after administration of vaccine will be reported as described below.

Definitions

Adverse Event
An AE is any untoward medical occurrence, including a dosing error, which may occur during or after vaccination.
The AE may or may not have a causal relationship with vaccination as indicated by physical signs, symptoms, and/or clinically significant laboratory abnormalities that occur. The definition includes intercurrent illnesses, injuries, exacerbation of pre-existing conditions, and events occurring as a result of product misuse or overdose.

A change in a laboratory variable is considered an AE if it leads to a change in the subject's functional status or if it is considered by the attending physician to be clinically significant. The following laboratory studies will be monitored during the course of this study (as previously outlined): full blood count (FBC), urea, creatinine, ALT, total bilirubin, albumin, alkaline phosphatase, sodium and potassium.

Adverse events will be graded and reported as detailed below.

Expected local reactions to the vaccine will not be recorded as an adverse event, but will be recorded on the CRFs. These include:

- Redness
- Swelling
- Pruritus
- Scaling
- Localised pain

Serious Adverse Event (SAE)
Any AE (whether or not considered related to the investigational product) that results in any of the following outcomes is defined as an SAE.

1) Death (i.e., results in death from any cause at any time)

2) Life-threatening event (i.e., the subject was, in the view of the investigator, at immediate risk of death from the event that occurred). This does not include an AE that, if it occurred in a more serious form, might have caused death.

3) Persistent or significant disability or incapacity (i.e., substantial disruption of one's ability to carry out normal life functions).

4) Hospitalisation: regardless of length of stay, even if the hospitalization is a precautionary measure for continued observation.
Hospitalisation (including inpatient or outpatient hospitalization for an elective procedure) for a pre-existing condition that has not worsened

unexpectedly does not constitute a serious AE.

5) An important medical event (that may not cause death, be life threatening, or require hospitalization) that may, based upon appropriate medical judgment, jeopardize the subject and/or require medical or surgical intervention to prevent one of the outcomes listed above. Examples of such medical events include allergic reaction requiring intensive treatment in an emergency room or clinic, blood dyscrasias, or convulsions that do not result in inpatient hospitalization.

6) Results in a congenital anomaly or birth defect.

Adverse Events Assessment

For every adverse event (AE), an assessment of the relationship of the event to the administration of the vaccine will be undertaken. An intervention-related AE refers to an AE for which there is a probable or definite relationship to administration of a vaccine. The investigator will make interpretation of the causal relationship of the intervention to the AE in question. This interpretation will be based on the type of event, the relationship of the event to the time of vaccine administration, and the known biology of the vaccine therapy. The following are guidelines for assessing the relationship of administration of one of the study vaccines to the AE

1. NO RELATIONSHIP:
   - No temporal relationship to study product; *and*
   - Alternate aetiology (clinical state, environmental or other interventions); *and*
   - Does not follow known pattern of response to study product
2. POSSIBLE:
   - Reasonable temporal relationship to study product; *or*
   - Event not readily produced by clinical state, environmental or other interventions; *or*
   - Similar pattern of response to that seen with other vaccines
3. PROBABLE:
   - Reasonable temporal relationship to study product; *and*
   - Event not readily produced by clinical state, environment, or other interventions *or*
   - Known pattern of response seen with other vaccines
4. DEFINITE:
   - Reasonable temporal relationship to study product; *and*
   - Event not readily produced by clinical state, environment, or other interventions; *and*
   - Known pattern of response seen with other vaccines

Every AE observed or reported from the day of injection [Day 0] through to the end of the trial (twelve months after injection) will be recorded on the CRF.

All AEs will be followed until resolution of the symptom or laboratory change occurs, or until a non-study related causality is assigned.

Serious Adverse Event (SAE) Reporting

In order to comply with current regulations on serious adverse event reporting to Health Authorities the investigator pledges to document accurately the event, to respect notification deadlines, to provide the Sponsor with all necessary information and, if requested by the sponsor, to give access to source documents. The investigator should complete the SAE form provided by the sponsor (Oxford University) and report any SAE within 24hours using the email address SAE@well.ox.ac.uk .

All SAEs will be reported by telephone, or email or fax to the local safety monitor and through her/him to the sponsor, the Research Ethics Committee of Oxford University and the Conseil National de Recherche en Santé, within one working day of the investigator becoming aware of the SAE occurrence.

Only suspected unexpected serious adverse reactions will be sent to the Medicines and Healthcare Products Regulatory Agency (MHRA) of the United Kingdom and this will be done within 7 days. The sponsor pledges to inform the Authorities of any trial discontinuation and specify the reason for discontinuation.

Adverse Event Monitoring

Local Safety Monitor

A Local Safety Monitor (LSM) will be appointed to provide real-time safety oversight. The LSM will review SAEs immediately after they occur and follow these events until resolution. The LSM has the power to terminate the study if deemed necessary following a SAE.

Reference safety parameters that are outcome measures (Section 10)

Methods and Timing for Assessing, Recording, and Analyzing Safety Parameters

The following scale will be used to assess the severity of all AEs:

0 = Absence of the indicated symptom
1 = Mild (awareness of a symptom but the symptom is easily tolerated)
2 = Moderate (discomfort enough to cause interference with usual activity)
3 = Severe (incapacitating; unable to perform usual activities; requires absenteeism or bed rest)
4 = Life-threatening

Reporting of Pregnancy

All subjects will be required to be using a reliable method of barrier contraception (i.e., condoms) prior to study enrolment, for the whole duration of the study and for 3 months after the last follow-up visit. A pregnancy test (urine) will be performed on each female subject on vaccination day. Should a subject become pregnant during the trial, she will be followed according to the protocol but in addition will be followed until pregnancy outcome.

Procedures to be Followed in the Event of Abnormal Laboratory Test Values or Abnormal Clinical Findings

Any subject with a clinically significant finding (e.g. hypertension) or abnormal biochemical or haematological may have the test repeated to ensure this was not a single occurrence prior to inclusion. If the test remains clinically significant the subject will be excluded from the study and referred to their HIV physician or GP as appropriate for further investigation and management with the permission of the volunteer. If occurring during the trial, tests may be repeated to verify the results, and further tests may be instigated to establish causality. The volunteer will be kept informed of all such occurrences and advised of actions to be taken, and their verbal/written consent taken for extra tests where appropriate.

Halting Rules

Any SUSAR will result in suspension of the trial until a safety review is convened.

9 CLINICAL MONITORING STRUCTURE

Local monitor: Prof Papa Salif Sow

Independent monitor: Ceri McKenna, Appledown

This section will describe the study monitoring to be conducted to ensure the safety and conduct of the study complies with 45 CFR 46, GCP and ICH Guidelines, DMID and other sponsor collaborator's guidelines, as appropriate.

Site Monitoring Plan

Set-up Visit

A set-up visit will be performed before the inclusion of the first subject in the study. The Monitor will verify and document that the material to be used during the trial has been received and that the investigational team has been properly informed about the trial, regulatory requirements, and the SOPs.

Follow-up Visits

The Monitor will carry out regular follow-up visits. The investigator will be available for these visits and to allow the monitoring staff direct access to subject medical file and CRFs. The Monitor is committed to professional secrecy.

During the visits, the Monitor will:

- carry out a quality control of trial progress: respect of protocol and operating guidelines, data collection, signature of consent forms, completion of document and appearance of SAE,
- sample and product management, cold chain monitoring,
- collect the CRFs and correspondent correction sheets,
- assess the inclusions in order to evaluate the number of complete or on-going observations.

The Monitor will discuss any problem with the investigator and define with him the actions to be taken.

Once the CRFs corresponding to the last visit have been returned duly completed and signed, the investigator must be available to complete the correction sheets transmitted by the Monitor, if necessary, until the database is locked.

Close-out Visit

A close-out visit will be performed at the end of the trial. Its goals are to make sure that:

- The centre has all the documents necessary for archiving,
- All paperwork is satisfactorily completed

Audits and Inspections

If necessary, a quality assurance audit will be carried out by independent auditors to make sure that the trial has been conducted according to the protocol and the applicable regulations.

An inspection may be conducted by Regulatory Authorities. The investigator must allow direct access to trial documents.

10 STATISTICAL CONSIDERATIONS

Determination of the Sample Size

This is an observational and descriptive safety study, where 24 subjects will be vaccinated. This sample size should allow determination of the magnitude of the outcome measures, especially of serious and severe adverse events, rather than aiming to obtain statistical significance.

Statistical Methods

The analysis will be only descriptive, as the sample size does not allow to perform comparison.

Data Management

The Principal Investigator will be the data manager with responsibility for receiving, entering, cleaning, querying, analysing and storing all data that accrues from the study. The data will be entered into the subjects' CRFs and may be transferred to a database.

11 SOURCE DOCUMENTS AND ACCESS TO SOURCE DATA/DOCUMENTS

The investigators will maintain appropriate medical and research records for this trial, in compliance with ICH E6 GCP and regulatory and institutional requirements for the protection of confidentiality of subjects. The principal investigator, co-investigator and clinical research nurses will have access to records.

The investigators will permit authorized representatives of the sponsor(s), and regulatory agencies to examine (and when required by applicable law, to copy) clinical records for the purposes of quality assurance reviews, audits and evaluation of the study safety and progress.

All protocol required information will be collected in Case Report Forms designed by the investigator. All source documents e.g., blood results, radiology reports, will be filed in the CRF.

12 QUALITY CONTROL AND QUALITY ASSURANCE

Data will be evaluated for compliance with protocol and accuracy in relation to source documents. The study will be conducted in accordance with procedures identified in the protocol. SOPs will be used at all clinical and laboratory sites. Regular monitoring and an independent audit will be performed according to GCP/ICH. Following written standard operating procedures, the monitors will verify that the clinical trial is conducted and data are generated, documented (recorded), and reported in compliance with the protocol, GCP, and the applicable regulatory requirements. The investigational site will provide direct access to all trial related sites, source data/documents, and reports for the purpose of monitoring and auditing by the sponsor, and inspection by local and regulatory authorities.

13 ETHICS/PROTECTION OF HUMAN SUBJECTS

This protocol will be approved by the Oxford Tropical Research Ethics Committee (OXTREC) and the Comité National d' Ethique before this study commences. This trial will be conducted in accordance with the current revision of the Declaration of Helsinki, or with the International Conference for Harmonisation Good Clinical Practice (ICH-GCP) regulations and guidelines, whichever affords the greater protection to the subject.

Informed Consent Process

Written informed consent and assent will be obtained from all participants, prior to screening. The consent form to be used will have been approved by Comité National d' Ethique, and OXTREC. Approved translations of consent forms will be available in French. For any participants without fluent French language skills, an interpreter (Wolof) will be obtained for the screening visit and all subsequent visits. The participants may withdraw consent at any time throughout the course of the trial. A copy of the informed consent document will be given to the participants for their records. The rights and welfare of the participants will be protected by emphasizing to them that the quality of their medical care will not be adversely affected if they decline to participate in this study.

Subject Confidentiality

All records will be kept in a locked filing cabinet, which is accessed only by the investigators and the study nurse(s). All computer entry and networking programs will be done with coded numbers and initials only. Only the investigators, the clinical monitor, the ethics committees and the CNRS (or any other regulatory agencies, at the request of the collaborator) will have access to the records. Photographs taken of vaccination sites (with the subject's written informed consent) will not include the subject's face and will be identified by the subject's 3-digit identification number only. Once developed, photographs will be stored as confidential records as above. This material may be shown to other professional staff or be used for educational purposes or be included in a scientific publication. Every effort will be taken to maintain confidentiality.

The study protocol, documentation, data and all other information generated will be held in strict confidence. No information concerning the study or the data will be released to any unauthorized third party, without prior written approval of the sponsor.

14 DATA HANDLING AND RECORD KEEPING

The principle Investigator will be the data manager with responsibility for receiving, entering, cleaning, querying, analysing and storing all data that accrues from the study. The data will be entered into the subjects' CRFs and may be transferred to a database. This includes safety data, laboratory data (both clinical and immunological) and outcome data.

Study Records Retention

Essential documents will be retained until at least 2 years after the last approval of a marketing application in an ICH region and until there are no pending or contemplated marketing applications in an ICH region or at least 2 years have elapsed since the formal discontinuation of clinical development of the investigational product. These documents will be retained for a longer period however if required by the applicable regulatory requirements or by an agreement with the sponsor. It is the responsibility of the sponsor to inform the investigator/institution as to when these documents no longer need to be retained

Protocol Deviations

The investigator will conduct the trial in compliance with the protocol agreed to by the sponsor and the regulatory authority and which was given approval by OXTREC. The investigator will sign the protocol to confirm agreement.

The investigator will not implement any deviation from or changes of the protocol without agreement by the sponsor and prior review and documented approval from OXTREC of an amendment, except where necessary to eliminate an immediate hazard(s) to trial subjects, or when the change(s) involve only logistical or administrative aspects of the trial (e.g. change in monitor(s), change of telephone number(s)).

The investigator, or person designated by the investigator, will document and explain any deviation from the approved protocol in the protocol deviation file.

The investigator may implement a deviation from, or a change of, the protocol to eliminate an immediate hazard(s) to trial subjects without prior OXTREC approval. As soon as possible, the implemented deviation or change, the reasons for it, and, if appropriate, the proposed protocol amendment(s) should be submitted:

A. to OXTREC for review and approval and, if required,
B. to the sponsor for agreement and , if required,
C. to the regulatory authority(ies).

15 REFERENCES

1. Dye C, S.S., Dolin P, Pathania V, Raviglione MC, *Global burden of Tuberculosis. Estimated incidence, prevalence and mortality by country.* JAMA, 1999. **282**(7): p. 677-686.
2. Corbett, E.L., et al., *The growing burden of tuberculosis: global trends and interactions with the HIV epidemic.* Arch Intern Med, 2003. **163**(9): p. 1009-21.
3. Dye, C., *Global epidemiology of tuberculosis.* Lancet, 2006. **367**: p. 938-940.
4. de Colombani, P., et al., *European framework to decrease the burden of TB/HIV.* Eur Respir J, 2004. **24**(3): p. 493-501.
5. Corbett, E.L., et al., *Tuberculosis in sub-Saharan Africa: opportunities, challenges, and change in the era of antiretroviral treatment.* Lancet, 2006. **367**(9514): p. 926-37.
6. Colditz GA, B.T., Berkey CS, Wilson ME, Burdick E, Fineberg HV, Mosteller F, *Efficacy of BCG vaccine in the prevention of tuberculosis: Meta-analysis of the published literature.* JAMA, 1994. **271**(9): p. 698-702.
7. Rodrigues LC, D.V., Wheeler JG, *Protective efficacy of BCG against tuberculous meningitis and miliary tuberculosis: a meta-analysis.* International Journal of Epidemiology 1993. **22**(6): p. 1154-1158.
8. Trunz, B.B., P. Fine, and C. Dye, *Effect of BCG vaccination on childhood tuberculous meningitis and miliary tuberculosis worldwide: a meta-analysis and assessment of cost-effectiveness.* Lancet, 2006. **367**(9517): p. 1173-80.
9. Murray CJL, S.J., *Modeling the impact of global tuberculosis control strategies.* PNAS, 1998. **95**: p. 13881-13886.
10. Ginsberg, A.M., *Tuberculosis vaccines: State of the Science.* NIAID Report, 2005.
11. Raviglione, M.C. and M.W. Uplekar, *WHO's new Stop TB Strategy.* Lancet, 2006. **367**(9514): p. 952-5.
12. WHO, *The global plan to Stop TB 2006-2015.* 2006.
13. Flynn, J.L. and J. Chan, *Immunology of tuberculosis.* Annu Rev Immunol, 2001. **19**: p. 93-129.
14. Schneider, J., et al., *Enhanced immunogenicity for CD8+ T cell induction and complete protective efficacy of malaria DNA vaccination by boosting with modified vaccinia virus Ankara.* Nat Med, 1998. **4**(4): p. 397-402.
15. McShane, H., et al., *Enhanced immunogenicity of CD4(+) t-cell responses and protective efficacy of a DNA-modified vaccinia virus Ankara prime-boost vaccination regimen for murine tuberculosis.* Infect Immun, 2001. **69**(2): p. 681-6.
16. Hanke, T., et al., *Effective induction of simian immunodeficiency virus-specific cytotoxic T lymphocytes in macaques by using a multiepitope gene and DNA prime-modified vaccinia virus Ankara boost vaccination regimen.* J Virol, 1999. **73**(9): p. 7524-32.
17. Horwitz, M.A., et al., *Protective immunity against tuberculosis induced by vaccination with major extracellular proteins of Mycobacterium tuberculosis.* Proc Natl Acad Sci U S A, 1995. **92**(5): p. 1530-4.
18. Launois, P., et al., *T-cell-epitope mapping of the major secreted mycobacterial antigen Ag85A in tuberculosis and leprosy.* Infect Immun, 1994. **62**(9): p. 3679-87.
19. Huygen, K., et al., *Mapping of TH1 helper T-cell epitopes on major secreted mycobacterial antigen 85A in mice infected with live Mycobacterium bovis BCG.* Infect Immun, 1994. **62**(2): p. 363-70.
20. Belisle, J.T., et al., *Role of the major antigen of Mycobacterium tuberculosis in cell wall biogenesis.* Science, 1997. **276**(5317): p. 1420-2.

21. Huygen, K., et al., *Immunogenicity and protective efficacy of a tuberculosis DNA vaccine.* Nat Med, 1996. **2**(8): p. 893-8.
22. Denis, O., et al., *Vaccination with plasmid DNA encoding mycobacterial antigen 85A stimulates a CD4+ and CD8+ T-cell epitopic repertoire broader than that stimulated by Mycobacterium tuberculosis H37Rv infection.* Infect Immun, 1998. **66**(4): p. 1527-33.
23. Smith, S.M., et al., *Human CD8+ CTL specific for the mycobacterial major secreted antigen 85A.* J Immunol, 2000. **165**(12): p. 7088-95.
24. Goonetilleke, N.P., et al., *Enhanced immunogenicity and protective efficacy against Mycobacterium tuberculosis of bacille Calmette-Guerin vaccine using mucosal administration and boosting with a recombinant modified vaccinia virus Ankara.* J Immunol, 2003. **171**(3): p. 1602-9.
25. Blanchard, T.J., et al., *Modified vaccinia virus Ankara undergoes limited replication in human cells and lacks several immunomodulatory proteins: implications for use as a human vaccine.* J Gen Virol, 1998. **79 (Pt 5)**: p. 1159-67.
26. Mayr, A., et al., *[The smallpox vaccination strain MVA: marker, genetic structure, experience gained with the parenteral vaccination and behavior in organisms with a debilitated defence mechanism (author's transl)].* Zentralbl Bakteriol [B], 1978. **167**(5-6): p. 375-90.
27. Sutter, G. and B. Moss, *Nonreplicating vaccinia vector efficiently expresses recombinant genes.* Proc Natl Acad Sci U S A, 1992. **89**(22): p. 10847-51.
28. Cosma, A., et al., *Therapeutic vaccination with MVA-HIV-1 nef elicits Nef-specific T-helper cell responses in chronically HIV-1 infected individuals.* Vaccine, 2003. **22**(1): p. 21-9.
29. Goonetilleke, N., et al., *Induction of multifunctional human immunodeficiency virus type 1 (HIV-1)-specific T cells capable of proliferation in healthy subjects by using a prime-boost regimen of DNA- and modified vaccinia virus Ankara-vectored vaccines expressing HIV-1 Gag coupled to CD8+ T-cell epitopes.* J Virol, 2006. **80**(10): p. 4717-28.
30. Dorrell, L., et al., *Expansion and diversification of virus-specific T cells following immunization of human immunodeficiency virus type 1 (HIV-1)-infected individuals with a recombinant modified vaccinia virus Ankara/HIV-1 Gag vaccine.* J Virol, 2006. **80**(10): p. 4705-16.
31. Moorthy, V.S., et al., *Safety and immunogenicity of DNA/modified vaccinia virus ankara malaria vaccination in African adults.* J Infect Dis, 2003. **188**(8): p. 1239-44.
32. Bejon, P., et al., *Safety profile of the viral vectors of attenuated fowlpox strain FP9 and modified vaccinia virus Ankara recombinant for either of 2 preerythrocytic malaria antigens, ME-TRAP or the circumsporozoite protein, in children and adults in Kenya.* Clin Infect Dis, 2006. **42**(8): p. 1102-10.
33. Webster, D.P., et al., *Safety of recombinant fowlpox strain FP9 and modified vaccinia virus Ankara vaccines against liver-stage P. falciparum malaria in non-immune volunteers.* Vaccine, 2006. **24**(15): p. 3026-34.
34. Meyer, R.G., et al., *A phase I vaccination study with tyrosinase in patients with stage II melanoma using recombinant modified vaccinia virus Ankara (MVA-hTyr).* Cancer Immunol Immunother, 2005. **54**(5): p. 453-67.
35. Smith, C.L., et al., *Recombinant modified vaccinia Ankara primes functionally activated CTL specific for a melanoma tumor antigen epitope in melanoma patients with a high risk of disease recurrence.* Int J Cancer, 2005. **113**(2): p. 259-66.
36. Taylor, J.L., et al., *Pulmonary necrosis resulting from DNA vaccination against tuberculosis.* Infect Immun, 2003. **71**(4): p. 2192-8.

37. McShane, H., et al., *Recombinant modified vaccinia virus Ankara expressing antigen 85A boosts BCG-primed and naturally acquired antimycobacterial immunity in humans*. Nat Med, 2004. **10**(11): p. 1240-4.
